# Physiologically based pharmacokinetic model of sodium-glucose cotransporter 2 inhibitors predicted pharmacokinetics and pharmacodynamics to explore dosage regimen for patients with type 2 diabetes mellitus and renal insufficiency

**DOI:** 10.3389/fphar.2025.1520268

**Published:** 2025-03-31

**Authors:** Guimu Guo, Meng Ke, Jianwen Xu, Wanhong Wu, Jiarui Chen, Chengjie Ke, Pinfang Huang, Cuihong Lin

**Affiliations:** ^1^ Department of Pharmacy, The First Affiliated Hospital of Fujian Medical University, Fuzhou, China; ^2^ Department of Pharmacy, National Regional Medical Center, Binhai Campus of the First Affiliated Hospital, Fujian Medical University, Fuzhou, China

**Keywords:** PBPKmodel, PD model, SGLT2 inhibitors, renal insufficiency, type 2 diabetes mellitus

## Abstract

**Objective:**

This study aimed to compare the hypoglycemic effects of four SGLT2 inhibitors (dapagliflozin, canagliflozin, empagliflozin, and ipragliflozin), simulate the 24-h urinary glucose excretion (UGE) of these inhibitors in T2DM patients with renal insufficiency, and investigate optimal dosage regimen for the SGLT2 inhibitor in these patients.

**Method:**

We established a physiologically based pharmacokinetic (PBPK) model of SGLT2 inhibitors using the PK-Sim software, and the renal physiological tissue structure was expanded to include renal tubules using the MoBi software. The PBPK/PD (pharmacodynamics) model of SGLT2 inhibitors was validated following comparison of the observed plasma concentration and pharmacokinetic parameters.

**Result:**

The model simulation results showed that 71.4% of the predicted pharmacokinetic parameters AUC (area under the curve) and C_max_ (peak concentration) closely matched the observed values within 0.8–1.3 folds accuracy. Further, 83.9% of the predicted concentration-time curves and 84.65% of the predicted 24-h urinary glucose excretion aligned with the observed data points within 0.5–2 folds accuracy. The MPE, AFE and AAFE values for all concentration-time data points were 0.90, 1.07 and 1.08, indicating that the predictive performance of the PBPK/PD model was robust and reliable. It was predicted that optimal hypoglycemic effects would be achieved in T2DM patients with mild, moderate, and severe renal insufficiency, when treated with ipragliflozin 50 mg qd, dapagliflozin 10 mg qd or canagliflozin 100 mg qd, empagliflozin 10 mg, respectively.

**Conclusion:**

This study provided a scientific basis for optimizing the dosage regimen in T2DM patients with renal insufficiency.

## 1 Introduction

The proximal convoluted tubule (PCT) in the kidney contains two sodium-glucose co-transporters, SGLT1 and SGLT2. These co-transporters are responsible for the reabsorption of approximately 99% of the glomerular filtered glucose. SGLT2 inhibitors are a new class of hypoglycemic agents that compete with glucose for the SGLT2 transporter to lower blood glucose levels, change threshold to concentrations, and enhance urinary glucose excretion (UGE) in patients with type 2 diabetes mellitus (T2DM) (Maurer et al., 2011). At present, a variety of SGLT2 inhibitors have been approved at home and abroad for managing blood glucose in patients with T2DM. For example, dapagliflozin, canagliflozin, and empagliflozin have been approved for market sale by the European Medicines Agency and the U.S. Food and Drug Administration. Ipragliflozin, luseogliflozin, and tofogliflozin also have been submitted to the Pharmaceuticals and Medical Devices Agency in Japan (Garcia-Ropero et al., 2018).

Diabetes mellitus (DM) is a common clinical disease, with 20%–40% of diabetic patients eventually developing chronic kidney disease (CKD). The kidneys of patients with CKD undergo structural and functional damage, and their glomerular filtration rate (GFR) progressively decreases (Devineni et al., 2015). Impaired renal function affects the pharmacokinetics (PK) and pharmacodynamics (PD) of SGLT2 inhibitors. Therefore, special attention should be given to their hypoglycemic effect and safety in patients with CKD and diabetic nephropathy (Khurana et al., 2015). Studies have shown that the area under the curve (AUC) of SGLT2 inhibitors in patients with T2DM and renal insufficiency increases as renal function declines, compared with patients with T2DM and normal renal function. For example, the AUC of canagliflozin increases by 15%, 29%, and 53% in patients with T2DM and mild, moderate, and severe renal insufficiency, respectively (Khurana et al., 2015). Similarly, ipragliflozin shows an increase in AUC of 40% and 47% in patients with T2DM and moderate and severe renal insufficiency, respectively (Scheen, 2015). The hypoglycemic effect of SGLT2 inhibitors decreases with declining renal function in patients with T2DM. For example, in patients with mild, moderate, and severe renal insufficiency, the UGE of dapagliflozin decreases by 42%, 83%, and 84%, respectively (Kasichayanula et al., 2013), while that of empagliflozin decreases by 37%, 43%, and 81%, respectively (Scheen, 2015).

Besides, there are significant differences in the efficacy of different SGLT2 inhibitors among patients with T2DM and normal renal function. Zaccardi et al. conducted a meta-analysis of 38 randomized controlled trials involving 23,997 participants to evaluate the efficacy and safety of SGLT2 inhibitors in adults with T2DM (Zaccardi et al., 2016). They found that, compared to other SGLT2 inhibitors (canagliflozin 100 mg, dapagliflozin 5 or 10 mg, and empagliflozin 10 or 25 mg), canagliflozin 300 mg significantly reduced HbA1c levels and fasting glucose in patients with T2DM. Similarly, Pinto et al. also concluded that canagliflozin 300 mg showed better efficacy than other SGLT2 inhibitors, empagliflozin 25 mg was more effective than dapagliflozin 10 mg, and empagliflozin (10 mg and 25 mg) showed similar efficacy to canagliflozin 100 mg (Pinto et al., 2022). However, to date, no literature or clinical studies have reported differences in efficacy among different SGLT2 inhibitors in patients with T2DM and renal insufficiency. Moreover, the application of SGLT2 inhibitors in patients with T2DM and renal insufficiency is limited. For example, dapagliflozin and empagliflozin are recommended for patients with eGFR >45 mL/min/1.73 m^2^, while canagliflozin can be used in patients with eGFR >30 mL/min/1.73 m^2^ (Grempler et al., 2012; Washburn and Poucher, 2013). Therefore, the dosage regimen of SGLT2 inhibitors in patients with T2DM and different degrees of CKD require further clarification.

In recent years, the PBPK model has developed rapidly in the academic and pharmaceutical fields, becoming integral to the drug development process (Kuepfer et al., 2016). The PBPK model demonstrates robust predictive ability by prospectively simulating concentration-time curves in the specific compartments. It quantitatively characterizes drug exposure at the site of action and investigates the pharmacological effects on specific organs or tissues. Initially established and verified in healthy subjects, the PBPK model then adjusts relevant physiological parameters for application in specific populations. This sequential approach yields PK/PD data for diverse populations. The extrapolation of the PBPK model transforms exploratory trials requiring a large number of subjects into confirmatory trials requiring only a small number of subjects. This approach improves trial efficiency and safety, reduces costs, shortens trial durations, and realizes the development of reasonably optimized dosage regimens under various pathological conditions (Perez-Urizar et al., 2000).

The purposes of this study were twofold. First, we aimed to construct a PBPK model for SGLT2 inhibitors combined with the mathematical model of UGE, and simulate and validate the PK and PD of SGLT2 inhibitors in both healthy subjects and T2DM patients with renal insufficiency. Second, we aimed to compare the hypoglycemic effects of four SGLT2 inhibitors (dapagliflozin 5 mg and 10 mg, canagliflozin 100 mg and 300 mg, empagliflozin 10 mg and 25 mg, and ipragliflozin 50mg and 100 mg), simulate the 24-h UGE of these inhibitors in patients with T2DM and renal insufficiency, and investigate optimal dosage regimen for the SGLT2 inhibitor in these patients.

## 2 Methods

### 2.1 The modeling process

**FIGURE 1:**
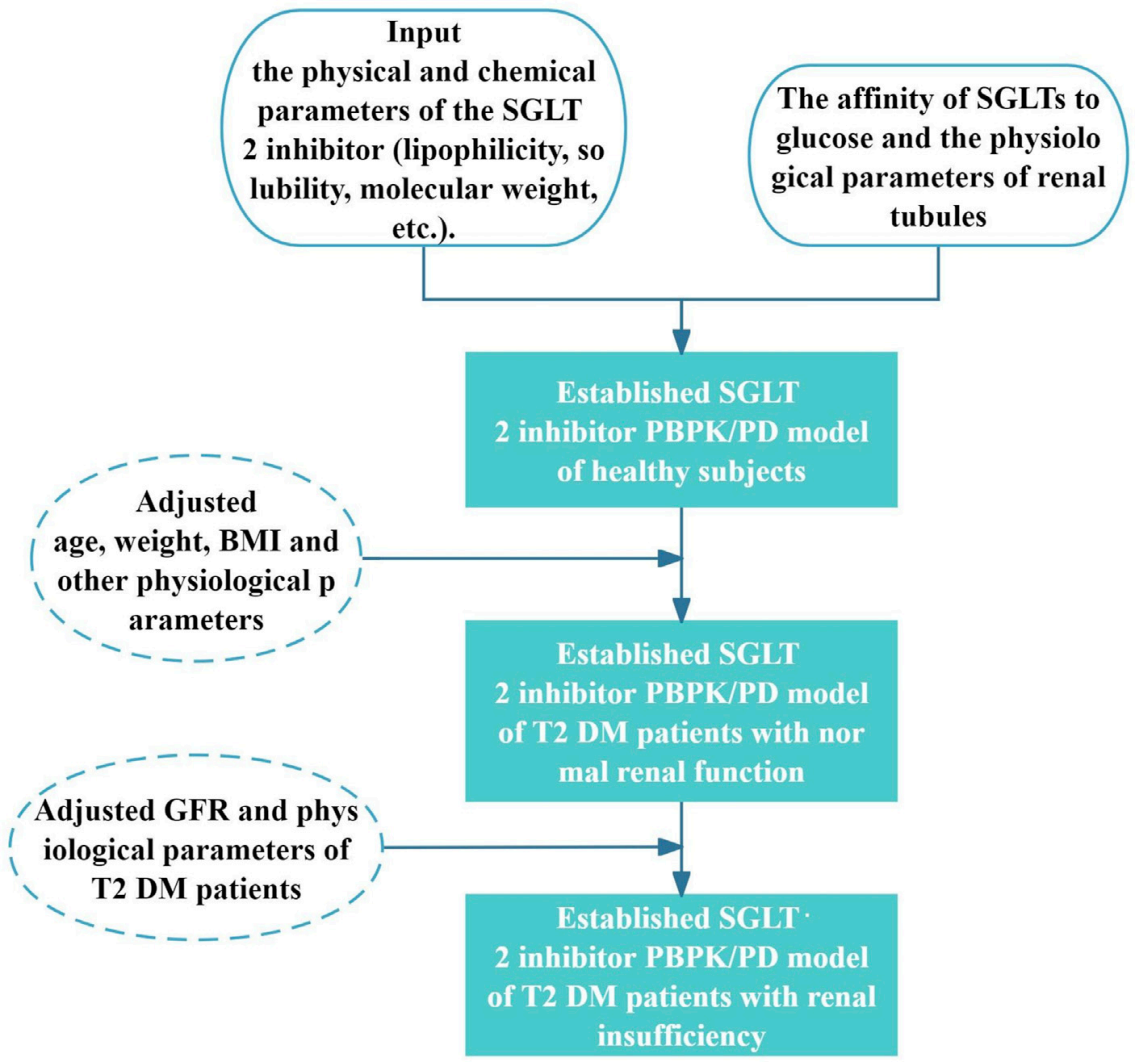
Flow chart of SGLT2 inhibitors PBPK/PD modeling construction.

First, using PK-Sim^®^ and MoBi^®^ version 11 software programs produced by Open Systems Pharmacology Suite/Leverkusen (Germany), we established the SGLT2-inhibitor PBPK/PD model in healthy subjects. Subsequently, age, weight, BMI, and other parameters were adjusted using the built-in algorithm of the PK-Sim software to obtain the physiological parameters of patients with T2DM, and establish the SGLT2-inhibitor PBPK/PD model of patients with T2DM and normal renal function. Then, by setting the GFR of virtual patients combined with the renal insufficiency tool in the PK-Sim software, the PBPK/PD model was extrapolated to patients with T2DM and renal insufficiency. The observed values validated the SGLT2-inhibitor PBPK/PD model. The clinically measured PK/PD data was obtained by GetData Graph Digitizer version 2.26 (GetData software development company, Montgomery St, Kogarah NSW, Australia) and plotted by OriginLab Origin^®^ 2024 (MicroCal, Northampton, Massachusetts, USA). The modeling process was shown in Figure 1.

### 2.2 Pharmacokinetic (PK)/pharmacodynamic (PD) data

We searched for scientific papers related to dapagliflozin, canagliflozin, empagliflozin, and ipragliflozin on the PubMed and Google Scholar websites, and information was collected on hypoglycemic therapy with SGLT2 inhibitors alone in patients with T2DM and healthy subjects. Specifically, we extracted: (1) number of subjects, therapeutic regimens, and doses; (2) plasma drug-time curve data points; (3) 24-h urinary glucose excretion; and (4) disease status (healthy or T2DM) and renal function status (Supplementary Table S1).

### 2.3 Establishment and validation the SGLT2-inhibitor PBPK model

Physicochemical parameters (lipophilicity, solubility, and molecular weight, among others) of the drug and the affinity parameters of SGLTs for glucose were obtained from the literature and DrugBank database and were input into the PK-Sim software to construct the SGLT2-inhibitor PBPK model (Drugbank, 2024; Drugbank, 2025a; Drugbank, 2025b; Drugbank, 2025c) (Table 1).

**TABLE 1:** PBPK modeling parameters of SGLT2 inhibitors.

Parameter	Dapagliflozin	Canagliflozin	Empagliflozin	Ipragliflozin	Glucose
MW(g/mol)	408.87 (Drugbank)	444.52 (Drugbank)	450.9 (Drugbank)	404.5 (Drugbank)	180.16 (Alkabbani and Gamble, 2021)
LogP	2.67 (2.52 (Drugbank))	3.89 (3.52 (Drugbank))	1.21 (1.79 (Devineni et al., 2015d)	2.40 (Drugbank)	−3.3 (Alkabbani and Gamble, 2021)
solubility (mg/mL)	0.22 (Callegari et al., 2021)	0.1 (Mori et al., 2016)	0.13 (Zhang et al., 2023)	0.0299 (Drugbank)	909 (Alkabbani and Gamble, 2021)
fup	0.09 (Alkabbani and Gamble, 2021)	0.017 (Mori et al., 2016)	0.14 (Zhang et al., 2023)	0.054 (Drugbank)	1 (Alkabbani and Gamble, 2021)
PKa	12.75 (Drugbank)	12.57 (Drugbank)	12.57 (Devineni et al., 2015d)	12.57 (Alkabbani and Gamble, 2021)	-
formulation	dissolved				-
Specific intestinal permeability (cm/min)	5.45*10^−5^ (Balazki et al., 2018)	0.02196 (optimized)	5.71*10^−4^ (optimized)	2.91*10^−5^ (optimized)	4.22*10^−7^ (Alkabbani and Gamble, 2021)
partition coefficients	Rodgers and Rowland				
cellular permeabilities	PK-Sim standard				
Specific organ permeability (cm/min)	1.13*10^−3^ (Balazki et al., 2018)	6.65*10^−5^ (optimized)	3.30*10^−6^ (optimized)	1.31*10^−4^ (optimized)	5.66*10^−10^ (Alkabbani and Gamble, 2021)
CL_H_ (L/h/kg)	-		0.09 (Y. Zhang et al., 2023)	0.16 (Veltkamp et al., 2011)	-
CL_UGT1A9_ (L/μmol/min)	0.40 (Callegari et al., 2021)	1.2 (optimized)	-	-	-
CL_UGT2B7_ (L/μmol/min)	6.60*10^−3^ (Callegari et al., 2021)	-	-	-	-
CL_UGT2B4_ (L/μmol/min)	-	0.14 (optimized)	-	-	-
CL_Other Enzyme_ (L/μmol/min)	0.14 (Callegari et al., 2021)	0.06 (optimized)	-	-	-
CL_R_(L/h)	0.79 (Jo et al., 2021)	-	-	-	-
GFR fraction	-	0.33 (Drugbank)	0.44 (0.286 (Devineni et al., 2015d)	0.014 (Alkabbani and Gamble, 2021)	-
P-gp K_m_ (μmol/L)	-	0.19 (optimized)	-	-	-
P-gp K_cat_ (min^-1^)	-	2.4 (optimized)	-	-	-
SGLT1 K_m_ (μmol/L)	-	-	-	-	400 (Yakovleva et al., 2019)
SGLT1 K_cat_ (min^-1^)	-	-	-	-	65000 (Yakovleva et al., 2019)
SGLT2 K_m_ (μmol/L)	-	-	-	-	2000 (Yakovleva et al., 2019)
SGLT2 K_cat_ (min^-1^)	-	-	-	-	17895.76 (Yakovleva et al., 2019)
K_d_ for SGLT1 (mol/L)	2.5 (Hummel et al., 2012)	1.25 (optimized)	15 (optimized)	5 (optimized)	-
K_off_ for SGLT1 (h^−1^)	1.1*10^−4^ (Hummel et al., 2012)^a^	1.1*10^−4^ (Hummel et al., 2012)^a^	1.1*10^−4^ (Hummel et al., 2012)^a^	1.1*10^−4^ (Hummel et al., 2012)^a^	-
K_d_ for SGLT2 (μmol/L)	1.57*10^−3^ (Balazki et al., 2018)	3.14*10^−3^ (optimized)	0.057 (Grempler et al., 2012)	6.93*10^−3^ (optimized)	-
K_off_ for SGLT2 (h^−1^)	6.3*10^−4^ (Balazki et al., 2018)^a^	6.3*10^−4^ (Balazki et al., 2018)^a^	0.6 (Grempler et al., 2012)	6.3*10^−4^ (Balazki et al., 2018)^a^	-
K_i_ for SGLT1 (nmol/L)	119.29 (Yakovleva et al., 2019)	770.5 (Yakovleva et al., 2019)	797.61(Y. Zhang et al., 2023)	860 (Washburn and Poucher, 2013)	-
K_i_ for SGLT2 (nmol/L)	0.1 (Yakovleva et al., 2019)	4 (Yakovleva et al., 2019)	0.64 (Y. Zhang et al., 2023)	2.8 (Washburn and Poucher, 2013)	-

^a^ This study assumed the same K_off_ values for the gliflozin class.

K_m_ was the Michaelis constant; K_cat_ = Vmax/transporter concentration; K_d_ was the association rate constant; K_off_ was the dissociation rate constant; Ki was the inhibition constant of the drug on the transporter; MW, was the molecular weight of the drug; lipophilicity was the lipophilicity of the drug; f_up_ was the percentage of unbound drug in plasma; PK_a_, was the acid dissociation constant; CL, was the clearance; CL_H_, was the hepatic clearance; CL_R_, was the renal clearance.

#### 2.3.1 The PBPK model of healthy subjects

The SGLT2 inhibitors were primarily metabolized into inactive products by UDP-glycosyltransferases (such as UGT1A9, UGT2B4, and UGT2B7), and a few SGLT2 inhibitors—such as dapagliflozin and canagliflozin—were metabolized into inactive products by cytochrome P450 enzymes (like CYP3A4, CYP2C9, CYP2D6, among others). The SGLT2 inhibitors and their inactive metabolites were mainly excreted in the feces and urine, with a small amount of the drug excreted in the urine as a prototype drug.

The modeling parameters from Mori K et al. and Callegari E et al. were incorporated into the PBPK model of dapagliflozin (Callegari et al., 2021; Mori et al., 2016). Further, we verified the reliability of the dapagliflozin PBPK model using the clinical measured data sets of single and multiple oral doses of 10 and 20 mg of dapagliflozin (Kasichayanula et al., 2014). According to the clinical pharmacokinetic studies in healthy subjects, the liver clearance (CL) values for empagliflozin and ipragliflozin were 0.09 and 0.16 L/h/kg, respectively (Veltkamp et al., 2011; Zhang et al., 2023). We combined the parameter identification modules of PK-Sim software with clinical measurement data sets to optimize the gastrointestinal permeability coefficients and cell permeability coefficients of empagliflozin and ipragliflozin.

Previous mass balance studies showed that canagliflozin is mainly metabolized via glucuronidation by UGT1A9 and UGT2B4 to two inactive O-glucuronide metabolites, and has only minor (7% in humans) metabolism by CYP3A4. For the contribution to glucuronidation, the relative contribution rates of UGT1A9 and UGT2B4 metabolism were 83% and 17%, respectively (Scheen, 2014). Utilizing the PK-Sim software and based on clinical data from Devineni D, et al., where healthy subjects were administered a 300 mg intravenous dose of canagliflozin, the total hepatic clearance was calculated to be 1.4 L/μmol/min (Devineni et al., 2015b). Subsequently, by integrating the relative contributions of hepatic enzymes and applying the formula CL_UGT1A9 or UGT2B4_ = hepatic clearance rate in healthy subjects * relative contribution rate of hepatic enzymes, the clearances for UGT1A9 and UGT2B4 were found to be 1.2 L/μmol/min and 0.14 L/μmol/min, respectively. The extra clearance rate, CL_Other Enzyme_, which represents the metabolic action of CYP3A4, is calculated using the formula CL_Other Enzyme_ = CL_healthy_ - CL_UGT1A9_ - CL_UGT2B4_, yielding a value of 0.06 L/μmol/min. In addition, canagliflozin was a P-glycoprotein (P-gp) substrate, based on clinical drug interaction study with cyclosporine, the K_m_ and K_cat_ values of P-gp were optimized to be 0.19 μmol/L and 2.4 min^−1^, respectively (Devineni et al., 2015c).

#### 2.3.2 The PBPK model of T2DM patient with normal renal function

The age, weight, and BMI of patients with T2DM and normal renal function were set according to the demographic characteristics of the patients in the clinical trial (Supplementary Table S1). Meanwhile, the corresponding physiological parameters of the virtual patients were generated using the built-in algorithm of the software to establish and verify the PBPK model of patients with T2DM.

#### 2.3.3 The PBPK model of patients with T2DM and renal insufficiency

After verifying the predictive performance of the PBPK model of SGLT2 inhibitors in healthy subjects and patients with T2DM and normal renal function, the corresponding GFR value was set according to the observed values of patients with renal insufficiency. The physiological parameters were generated by the built-in database of the model, and the PBPK model was extrapolated to the population of patients with T2DM and renal insufficiency. Subsequently, the PBPK model was validated using clinically measured data.

### 2.4 Establishment and validation the SGLT2-inhibitor PD model

In patients with T2DM, glucose that remained after glomerular filtration was distributed along the proximal tubules, and the excess glucose that accumulated in the bladder was subsequently excreted in the urine (Zhang et al., 2023). Using MoBi software, the physiological structure of the kidney tissue was extended to include the renal tubules, which were divided into three segments: the proximal renal tubules (S1), the distal renal tubules and collecting tubules (S2), and the bladder lumen (S3). The PD model of SGLT2 inhibition incorporated the physiological parameters related to renal tubules (Supplementary Table S2) and used Supplementary Equations S1–S7 to describe the changes in glucose concentration in the proximal renal tubule and to calculate the urinary glucose excretion over time (Wang et al., 2022). The detailed modeling process for the PD model is described in the “supplementary methods” section of the supplementary materials.

The predicted drug-concentration-time curve was compared to the observed data set, and the fold error between the predicted and observed values of the PK parameters (AUC, C_max_) was obtained. Subsequently, the predictive performance of the PBPK/PD model for SGLT2 inhibitors was comprehensively evaluated. The fold error was calculated was calculated by Equation 1. If the fold error was between 0.5 and 2.0, the predictive performance of the model was considered acceptable (Huang and Rowland, 2012). In addition, Equations 2–4 were used to calculate the mean prediction error (MPE), the average folding error (AFE) and the absolute average folding error (AAFE) of all concentration-time data points to assess the precision and the bias of the model.

$$F\text{old error}=\frac{P\text{redicted}}{\text{Observed}}$$ (1)

$$\text{MPE}=\frac{1}{n}\sum \frac{\left(\text{predicted}-\text{observed}\right)}{\text{observed}}$$ (2)

$$\text{AFE}=10^{\frac{1}{n}\sum \log\left(\frac{\text{predicted}}{\text{observed}}\right)}$$ (3)

$$A\text{AFE}=10^{\frac{1}{n}\sum \left|\text{lo}g\left(\frac{\text{predicted}}{\text{observed}}\right)\right|}$$ (4)

where “Predicted” represents the predicted value and “Observed” represents the clinically measured value or the observed value.

### 2.5 Based on the PBPK/PD model of SGLT2-inhibitor to explore dosage regimen in patients with renal insufficiency

The PBPK/PD model of SGLT2 inhibitors was used to predict 24 h urinary glucose excretion in patients with T2DM and renal insufficiency following administration of the daily dose of each SGLT2 inhibitor (dapagliflozin 5 mg and 10 mg, canagliflozin 100 mg and 300 mg, empagliflozin 10 mg and 25 mg, and ipragliflozin 50 mg and 100 mg). After comparing the four SGLT2 inhibitors, the hypoglycemic effects were used to determine the recommended optimal dosage regimen for patients with T2DM and renal insufficiency. Considering the 30-year-old European T2DM virtual patient population as an example, the simulated sample was set to 100 people, 50% of whom were men, and the average GFR of patients with normal renal function and mild, moderate, and severe renal insufficiency were 105, 75, 45, and 15 mL/min, respectively.

### 2.6 Population simulation

The influence of individual variations in the population physiological parameters on the simulation results were investigated using the PK-Sim population simulation block. The demographic characteristics of the virtual population were consistent with clinically measured data from the literature, and the individual parameters of the virtual subjects were randomly generated using the distribution range defined by the PK-Sim software. The simulated sample was set to 1,000 people, 50% of whom were male. The demographic characteristics of the simulated population are shown in Supplementary Table S1. The dosage regimen of the virtual subjects was consistent with that reported in the literature (Veltkamp et al., 2011; Zhang et al., 2023). The predictive performance of the model was further validated by evaluating whether the clinically measured values fell within the 90% probability lines of the predicted drug concentration-time curve of the model.

## 3 Results

### 3.1 Establishment and validation of the SGLT2-inhibitor PBPK model

#### 3.1.1 The PBPK model of healthy subjects and patients with T2DM and normal renal function

**FIGURE 2:**
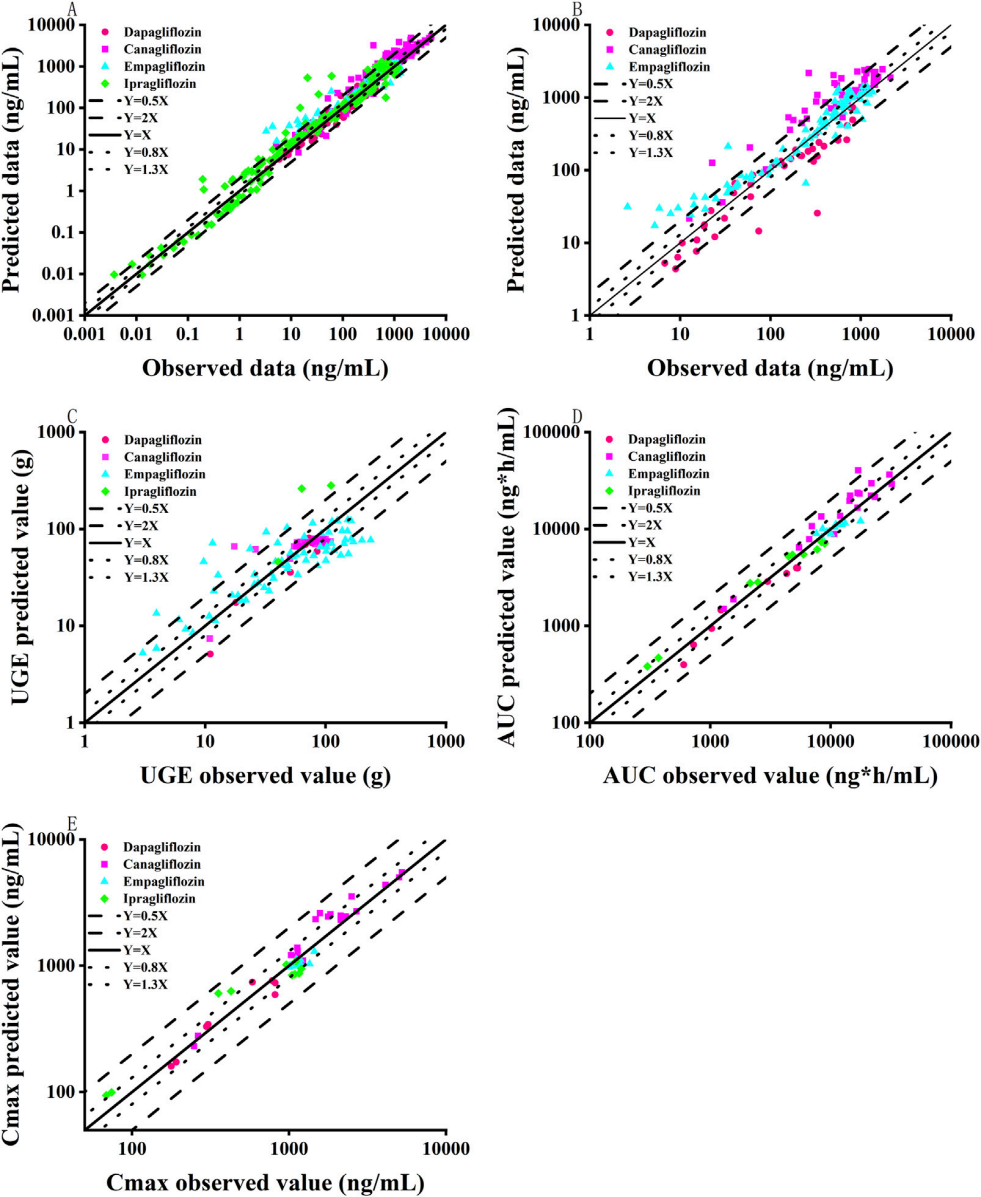
Goodness-of-fit plot of SGLT2 inhibitor PBPK/PD model predictions with measured data points. **(A)** Comparison of drug concentration-time curve fitting in patients with normal renal function; **(B)** Comparison of drug concentration-time curve fitting in patients with renal insufficiency; **(C)** Urinary glucose excretion; **(D)** Pharmacokinetic parameter AUC; **(E)** Pharmacokinetic parameter C_max_; Black solid line represents multiple error of 1-fold; Blue dotted line represents 0.8–1.3 folds; Black lines represent 0.5-2 folds.

This study successfully established and validated PBPK models for dapagliflozin, canagliflozin, empagliflozin, and ipragliflozin. The results showed that 86.5% of the predicted concentration-time curves between the clinical measured data points were within 0.5–2 folds (Figure 2A), and 76.8% of the predicted pharmacokinetic parameters (AUC, C_max_) of the SGLT2 inhibitors between the observed values were within 0.8–1.3 folds (Figures 2D, E; Table 2). The MPE, AFE and AAFE values for concentration-time data points in healthy subjects and patients with T2DM and normal renal function were 0.73, 1.11 and 1.16, respectively.

**TABLE 2:** Comparison of predicted and observed pharmacokinetic parameters in healthy subjects and T2DM patients with normal renal function.

Drug	References	Dosage regimen	AUC (ng*h/mL)			Cmax (ng/mL)		
			observed	predicted	fold error	observed	predicted	fold error
Dapagliflozin	Kasichayanula et al. (2011)	Po 10 mg SAD	602.00	397.62	0.66	177.83	159.92	0.90
		Po 20 mg SAD	1,027.00	937.74	0.91	298.00	329.92	1.11
		Po 10 mg MAD	727.00	637.94	0.88	191.00	172.36	0.90
		Po 20 mg MAD	1,225.00	1,452.65	1.19	305.00	342.67	1.12
Canagliflozin	Devineni, Murphy, et al. (2015)	Po 300 mg SAD	17375.00	23123.26	1.33	2,504.00	3,537.86	1.41
	Chen et al. (2015)	Po 100 mg SAD	10680.02	8,859.76	0.83	1,128.84	1,389.80	1.23
		Po 300 mg SAD	32131.77	29253.71	0.91	4,118.95	4,377.42	1.06
	Iijima et al. (2015)	Po 25 mg SAD	1,295.00	1,496.56	1.16	248.00	230.28	0.93
		Po 100 mg SAD	5,468.00	6,455.97	1.18	1,126.00	1,180.77	1.05
		Po 200 mg SAD	11991.00	13673.00	1.14	2,304.00	2,456.22	1.07
		Po 400 mg SAD	21836.00	29616.77	1.36	5,028.00	5,018.00	1.00
		Po 25 mg MAD	1,556.00	1877.12	1.21	263.00	277.96	1.06
		Po 100 mg MAD	6,635.00	7,853.33	1.18	1,136.00	1,313.79	1.16
		Po 200 mg MAD	16716.00	16587.43	0.99	2,688.00	2,695.75	1.00
		Po 400 mg MAD	30766.00	36430.92	1.18	5,236.00	5,505.95	1.05
	Inagaki et al. (2014)	Po 100 mg SAD	6,977.50	10710.15	1.53	1,226.33	1,097.42	0.89
		Po 200 mg SAD	14500.88	22087.38	1.52	2,133.60	2,303.73	1.08
Ipragliflozin	Veltkamp et al. (2011)	Po 5 mg SAD	300.58	382.53	1.27	68.47	94.01	1.37
		Po 30 mg SAD	2,145.75	2,760.17	1.29	354.64	603.34	1.70
		Po 100 mg SAD	7,727.49	6,141.09	0.80	960.91	1,018.76	1.06
		Po 5 mg MAD	370.78	468.30	1.26	74.06	99.39	1.34
Ipragliflozin	Veltkamp et al. (2011)	Po 30 mg MAD	2,487.51	2,810.78	1.13	427.01	627.39	1.47
		Po 100 mg MAD	8,924.35	7,063.53	0.79	1,105.30	1,082.73	0.98
	(W. Zhang et al., 2013)	Po 100 mg SAD	8,400.97	7,322.62	0.87	1,198.72	946.81	0.79
Empagliflozin	Friedrich et al. (2013)	Po 50 mg SAD	9,300.00	9,352.98	1.01	1,450.00	1,297.20	0.89
	Seman et al. (2013)	Po 50 mg SAD	8,580.00	10052.71	1.17	1,110.00	1,121.43	1.01
	Brand et al. (2012)	Po 50 mg SAD	8,430.00	8,179.91	0.97	1,180.00	1,112.63	0.94
	Zhao et al. (2015)	Po 25 mg SAD	10047.02	8,894.20	0.89	1,075.50	1,006.10	0.94

SAD, single dose; MAD, multiple dose; CKD, chronic kidney disease; Po: oral; T2DM, patients with type 2 diabetes; AUC, area under the concentration-time curve; C_max_, peak concentration; T_max_, time to peak.

#### 3.1.2 The PBPK model of patients with T2DM and renal insufficiency

After verifying the predictive performance of the SGLT2 inhibitor PBPK model in healthy subjects and patients with T2DM and normal renal function, the PBPK model was extrapolated to patients with T2DM and renal insufficiency by adjusting the GFR and physiological parameters of virtual T2DM patients according to clinical-patient mean values. As shown in Figure 2B and Table 3, 80.2% of the predicted concentration-time curves between the clinically measured data points were within 0.5–2 folds, and 64.3% of the predicted SGLT2-inhibitor pharmacokinetic parameters (AUC, C_max_, etc.) between the measured values were within 0.8–1.3 folds. In patients with T2DM and renal insufficiency, the MPE, AFE and AAFE values for concentration-time data points were 0.90, 1.07 and 1.08, respectively.

**TABLE 3:** Comparison of predicted and observed pharmacokinetic parameters in subjects with normal renal function and T2DM patients with renal insufficiency.

Drug	Reference	Dosage regimen	AUC (ng*h/mL)			Cmax (ng/mL)		
			observed	predicted	fold error	observed	predicted	fold error
Dapagliflozin	Kasichayanula et al. (2014)	po 50 mg SAD for T2DM with normal renal function	2,994.78	2,865.89	0.96	584.68	741.12	1.27
		po 50 mg SAD for mild CKD T2DM	4,313.79	3,466.97	0.80	782.05	760.95	0.97
		po 50 mg SAD for moderate CKD T2DM	5,322.10	3,940.57	0.74	815.23	731.51	0.90
		po 50 mg SAD for severe CKD T2DM	5,204.24	3,956.46	0.76	815.23	589.54	0.72
Canagliflozin	Devineni et al. (2015a)	po 200 mg SAD for subjects with normal renal function	14345.00	19548.51	1.36	1,475.00	2,335.53	1.58
		po 200 mg SAD for subjects with mild CKD	16719.00	23569.21	1.41	1,574.00	2,607.86	1.66
		po 200 mg SAD for subjects with moderate CKD	23311.00	21423.59	0.92	1773.00	2,449.51	1.38
		po 200 mg SAD for subjects with severe CKD	21596.00	22050.24	1.02	1834.00	2,557.71	1.39
	Inagaki et al. (2014)	Po 100 mg SAD for moderate CKD T2DM	8,355.80	13461.83	1.61	1,029.58	1,214.69	1.18
		Po 200 mg SAD for moderate CKD T2DM	16916.42	40351.40	2.39	2,133.60	2,490.23	1.17
Ipragliflozin	Ferrannini et al. (2013)	po 50 mg SAD for mild CKD T2DM	4,821.00	5,400.46	1.12	1,045.00	842.42	0.81
		po 50 mg SAD for moderate CKD T2DM	4,482.00	5,221.20	1.16	1,089.00	855.63	0.79
		po 50 mg SAD for severe CKD T2DM	5,948.00	5,470.78	0.92	1,161.00	869.59	0.75
Empagliflozin	Sarashina et al. (2014)	po 25 mg SAD for T2DM with normal renal function	7,589.41	8,998.65	1.19	1,002.54	995.59	0.99
		po 25 mg SAD for mild CKD T2DM	9,730.00	9,411.87	0.97	1,030.00	1,004.71	0.98
		po 25 mg SAD for moderate CKD T2DM	10800.00	10365.59	0.96	1,000.00	973.72	0.97
		po 25 mg SAD for severe CKD T2DM	12200.00	11287.72	0.93	1,070.00	993.81	0.93
Empagliflozin	Macha et al. (2014)	po 50 mg SAD for subjects with normal renal function	11006.07	11134.13	1.01	1,178.23	1,029.51	0.87
		po 50 mg SAD for subjects with mild CKD	13200.81	11484.10	0.87	1,353.31	1,035.84	0.77
		po 50 mg SAD for subjects with moderate CKD	12333.63	11109.49	0.90	1,178.23	1,015.86	0.86
		po 50 mg SAD for subjects with severe CKD	17700.00	12076.93	0.68	1,178.23	1,034.50	0.87

SAD, single dose; MAD, multiple dose; CKD, chronic kidney disease; Po: oral; T2DM, patients with type 2 diabetes; AUC, area under the concentration-time curve; C_max_, peak concentration; T_max_, time to peak.

The performance of the model, extraction of measured data, physiological changes in the process of drug administration, and accuracy of the sampling time points may have affected the prediction results. Overall, the predicted concentration-time curves were close to the observed values; specifically, 71.4% of the predicted pharmacokinetic parameters (AUC, C_max_) between the clinically measured values were within 0.8–1.3 folds, and 84.65% of the predicted concentration-time curves between the clinically measured data points were within 0.5–2 folds. The MPE, AFE and AAFE values for all concentration-time data points were 0.90, 1.07 and 1.08. Therefore, the predictive performance of the SGLT2-inhibitor PBPK/PD model was acceptable.

### 3.2 Establishment and validation of the SGLT2-inhibitor PD model

The SGLT2-inhibitor PD model was established and validated in healthy subjects and normal and renal impaired patients with T2DM. As shown in Figure 2C, 80.2% of the predicted 24-h urinary glucose excretion values were within 0.5–2 folds. The MPE, AFE and AAFE values for 24-h urinary glucose excretion values were −0.11, 0.76 and 1.41, respectively.

### 3.3 Based on the PBPK/PD model of SGLT2-inhibitor to explore dosage regimen in patients with renal insufficiency

**FIGURE 3:**
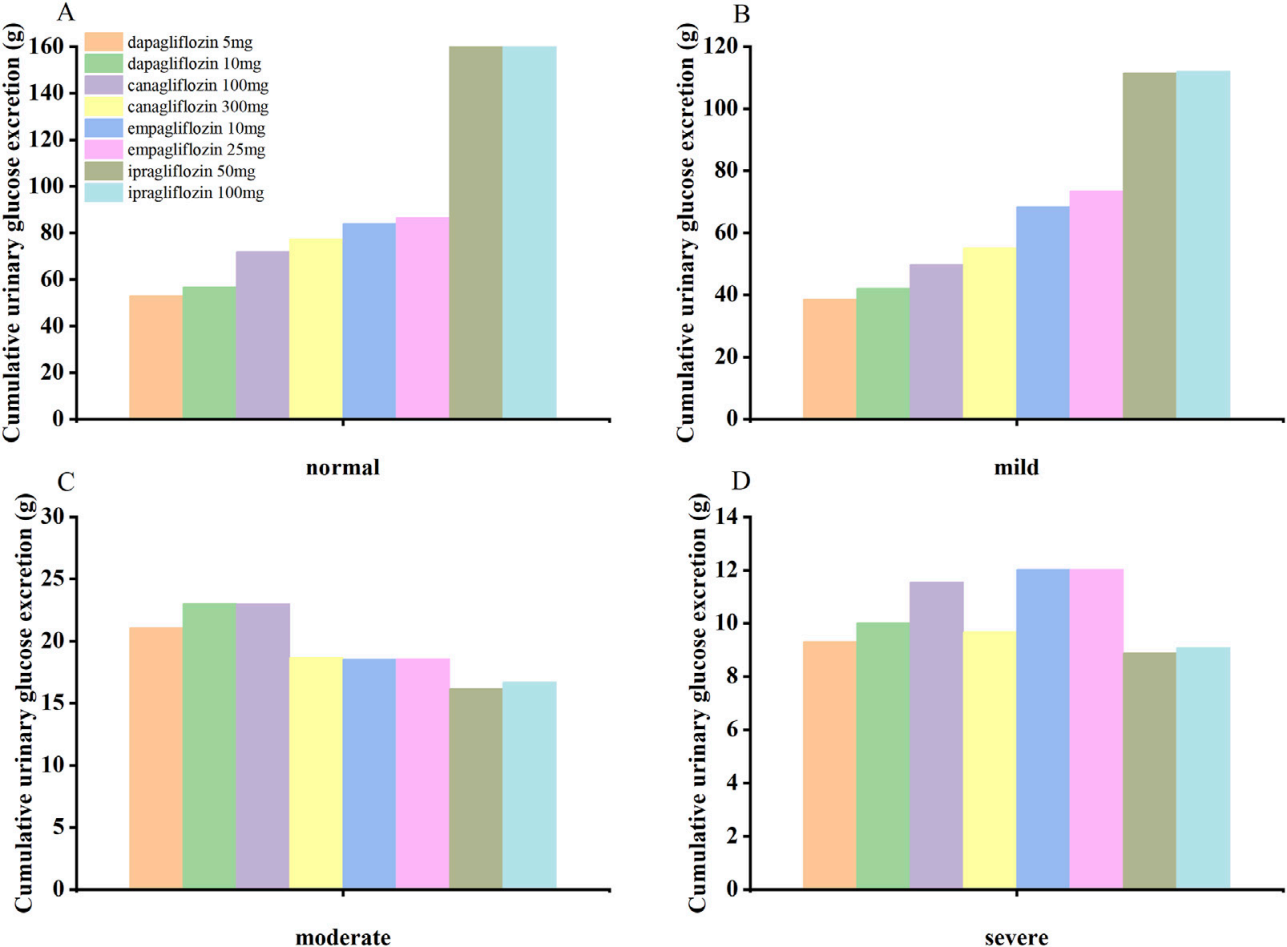
The SGLT2 inhibitor PBPK/PD model predicted the cumulative 24-hour urinary glucose excretion in T2DM patients with normal renal function **(A)** and mild **(B)**, moderate **(C)**, and severe **(D)** renal insufficiency.

The SGLT2-inhibitor PBPK/PD model was used to predict the concentration-time curve and UGE in patients with T2DM and normal renal function or renal insufficiency. The SGLT2-inhibitor PD model predicted the 24-h UGE in patients with T2DM using the daily dose of SGLT2 inhibitors, as shown in Figure 3 and Table 4. The hypoglycemic effects of the four SGLT2 inhibitors were as follows: (1) ipragliflozin 50 mg qd and 100 mg qd > empagliflozin 25 mg qd > empagliflozin 10 mg qd > canagliflozin 300 mg qd > canagliflozin 100 mg qd > dapagliflozin 10 mg qd > dapagliflozin 5 mg qd in T2DM patients with normal renal function; (2) ipragliflozin 50 mg qd and 100 mg qd > empagliflozin 25 mg qd > empagliflozin 10 mg qd > canagliflozin 300 mg qd > canagliflozin 100 mg qd > dapagliflozin 10 mg qd > dapagliflozin 5 mg qd in T2DM patients with mild renal insufficiency; (3) dapagliflozin 10 mg qd > canagliflozin 100 mg qd > dapagliflozin 5 mg qd > canagliflozin 300 mg qd > empagliflozin 10 mg qd and 25 mg qd > ipragliflozin 50mg and 100 mg qd in patients with T2DM and moderate renal insufficiency; (4) empagliflozin 10 mg qd and 25 mg qd > canagliflozin 100 mg qd > dapagliflozin 10 mg qd > canagliflozin 300 mg qd > dapagliflozin 5 mg qd > ipragliflozin 100 mg qd > ipragliflozin 50 mg qd in patients with T2DM and severe renal insufficiency.

**TABLE 4:** Based on the PBPK/PD model of SGLT2 inhibitors predicted the 24-h urinary glucose excretion in type 2 diabetic patients with renal insufficiency.

SGLT2 inhibitors	dosage	24 h urinary glucose excretion in patients with renal insufficiency, g			
		Normal (90%CI)	Mild (90%CI)	Moderate (90%CI)	Severe (90%CI)
dapagliflozin	5 mg	53.09 (40.91–69.68)	38.56 (29.16–46.80)	21.07 (11.69–32.40)	9.31 (4.59–16.28)
	10 mg	56.76 (44.41–73.44)	42.20 (32.41–50.62)	23.03 (13.44–34.36)	10.02 (5.16–16.99)
canagliflozin	100 mg	71.92 (51.06–93.04)	49.75 (0.05–120.46.87)	22.98 (10.90–39.62)	11.55 (6.97–19.40)
	300 mg	77.29 (57.04–99.15)	55.27 (6.10–125.59)	18.68 (7.26–35.38)	9.68 (5.80–16.48)
empagliflozin	10 mg	84.05 (26.00–165.05)	68.36 (44.96–85.10)	18.55 (5.69–38.97)	12.02 (5.27–24.79)
	25 mg	86.47 (26.11–165.44)	73.38 (46.22–92.15)	18.58 (5.70–39.08)	12.03 (5.29–24.80)
ipragliflozin	50 mg	192.09 (4.66–487.47)	111.47 (46.71–173.77)	16.15 (2.06–43.43)	8.88 (5.04–15.61)
	100 mg	192.71 (6.17–487.63)	111.97 (47.44–174.22)	16.69 (2.22–44.64)	9.08 (5.05–15.94)

### 3.4 Population simulation

According to the demographic characteristics of the clinical patients and the dosage regimen, the PBPK/PD model of SGLT2 inhibitors simulated the concentration-time curve and 24-h UGE in patients with T2DM. As shown in Supplementary Figures S1–S10, 81% of the clinically measured points in the concentration-time curve and 85% of the observed values of UGE were within the 90% confidence interval of the virtual population. The simulation results suggested that the PBPK/PD model of SGLT2 inhibitors had a high predictive performance.

## 4 Discussions

Studies have shown that SGLT2 inhibitors could benefit patients with CKD and show promise for treating patients with T2DM and renal insufficiency (Fioretto et al., 2018; Mende, 2022). However, the full potential of SGLT2 inhibitors in CKD remains underexplored. The differences in the efficacy of different SGLT2 inhibitors in patients with T2DM and renal insufficiency have not been reported. In this study, we constructed a PBPK model of SGLT2 inhibitors, combined with a mathematical model of UGE, to simulate and predict changes in the PK and PD of SGLT2 inhibitors in healthy subjects and patients with T2DM and renal insufficiency. We recommended dosage regimens for patients with renal insufficiency to achieve the lowest dose and the maximum therapeutic effect from the perspective of quantitative system pharmacology. It was predicted that optimal hypoglycemic effects would be achieved in T2DM patients with mild, moderate, and severe renal insufficiency, when treated with ipragliflozin 50 mg qd, canagliflozin 100 mg qd or dapagliflozin 10 mg qd, empagliflozin 10 mg, respectively. The PBPK/PD model of the SGLT2 inhibitor simulation results further verified the conclusion of clinical studies by Pinto et al. (2022), Zaccardi et al. (2016). This result provides guidance for selecting SGLT2 inhibitors in patients with T2DM and renal insufficiency, however, it still needs to be verified in controlled clinical trials.

Hummel et al. reported the binding and dissociation rate constants (K_d_ and K_off_) values of Dapagliflozin for SGLT1 and SGLT2. Grempler et al. studied the inhibitory efficacy and selectivity of different SGLT2 inhibitors on the transporter SGLTs (Grempler et al., 2012; Hummel et al., 2012). The selectivity affinity of canagliflozin, empagliflozin, and ipragliflozin for SGLT1 was 0.5 times, 6 times, and 2 times that of dapagliflozin, respectively, and canagliflozin and ipragliflozin were more inhibitory than dapagliflozin against SGLT2 with 2 and 4.4 times stronger inhibition strengths, respectively. The SGLT1 K_d_ values of canagliflozin, empagliflozin, and ipragliflozin were set to 1.25 mol/L, 15 mol/L, and 5 mol/L, respectively. The SGLT2 K_d_ values of canagliflozin and ipragliflozin were set to 3.14 * 10^–3^ μmol/L and 6.93 * 10^–3^ μmol/L, respectively (Table 1). The PD model of empagliflozin overestimated urinary glucose excretion in patients with T2DM 1–3 h after 25 mg empagliflozin administration, which may be due to differences in the *in vivo* and *in vitro* binding rate constants of empagliflozin to the SGLT2 transporter (Grempler et al., 2012).

Evidence supporting the use of SGLT2 inhibitors in patients with severe CKD is limited due to increased risks of adverse reactions like diabetic ketoacidosis, hypovolemia, and acute kidney injury in diabetic patients, making it often contraindicated in those with severe renal insufficiency (GFR <30 mL/min/1.73 m^2^) (Qiu et al., 2021). Nonetheless, a number of published controlled clinical trials have confirmed that CKD patients show good tolerance to SGLT2 inhibitors, such as, CREDENCE (ClinicalTrials.gov: NCT02065791), DAPA-CKD (ClinicalTrials.gov: NCT03036150), EMPA-KIDNEY (ClinicalTrials.gov: NCT03594110), significantly delaying the onset and development of diabetic nephropathy, reducing mortality from end-stage renal disease, and showing sustained benefits with long-term treatment (Heerspink et al., 2020; Perkovic et al., 2019; The et al., 2023). In our study, using a PBPK/PD model of SGLT2 inhibitors, the PK/PD of patients with T2DM and severe renal insufficiency were analyzed and compared for efficacy. We propose that in urgent cases where the use of SGLT2 inhibitors is necessary, empagliflozin 10 mg qd should be considered to achieve an optimal hypoglycemic effect.

An interesting finding from this study is that the decreased reabsorption capacity of SGLT transporters is a significant factor contributing to the reduced efficacy of SGLT2 inhibitors in patients with T2DM and renal insufficiency. Using the PBPK/PD model of SGLT2 inhibitors, our study analyzed the reabsorption capacity of the SGLT transporter system in patients with CKD and found that in patients with T2DM and moderate or severe renal insufficiency, the reabsorption capacity of the SGLT1 transporter decreased by 15% and 50%, respectively, while that of the SGLT2 transporter decreased by 60% and 90%, respectively (see Supplementary Table S3). Nakamura et al. findings also suggest that the impaired reabsorption capacity of the SGLT system limits the efficacy of SGLT2 inhibitors (Nakamura et al., 2004). They conducted a rat study and found significant reductions in the V_max_ values of both SGLT1 and SGLT2 in isolated renal apical membrane vesicles from rats subjected to 5/6 nephrectomy compared with the control group. Specifically, SGLT1 and SGLT2 V_max_ values were reduced by 56.7% and 47.8%, respectively, while the K_m_ value was not significantly changed.

Moreover, we found that the degree of decrease in the reabsorption capacity of the SGLT2 transporter in patients with renal insufficiency was similar to the degree of decrease in the UGE at 24 h after SGLT2 inhibitors treatment. For example, the 24 h UGE with treatment of dapagliflozin was decreased by 42%, 83%, and 84% in patients with mild, moderate, and severe renal insufficiency, respectively (Kasichayanula et al., 2013), further supporting the notion that reduced efficacy of SGLT2 inhibitors in patients with renal insufficiency correlates with impaired SGLT2 transporter function. In conclusion, our results suggested that the function of SGLT2 transporter may change in T2DM co-occurring with renal insufficiency, thus contributing to the reduced efficacy of SGLT2 inhibitors in this population.

The predictive performance of the PD model for SGLT2 inhibitors established in this study still requires improvement. Firstly, the model relied on manually inputting the average glucose level of patients and could not dynamically simulate changes in glucose and insulin levels following food intake nor accurately predict the decline in average blood glucose after SGLT2 inhibitor treatment. Secondly, it was unable to reliably predict the changes in blood glucose within 24 h. Thirdly, the liver and kidneys are interlinked in drug metabolism and excretion processes. Renal impairment can indirectly influence hepatic metabolic capabilities, affecting factors such as drug protein binding and the expression of hepatic enzymes. Our model of SGLT2 inhibitors for patients with renal impairment has not yet accounted for these factors (Butrovich et al., 2022). Furthermore, the clinically measured data used to validate the PK/PD model in patients with renal insufficiency were limited, especially in the PD model. More clinically measured data are needed to further validate the reliability of the model.

## 5 Conclusion

We successfully established the PBPK/PD model of SGLT2 inhibitors to simulate the PK and PD in healthy people and T2DM patients with renal insufficiency. The PBPK model simulated 24-h urinary glucose excretion in patients with renal insufficiency to compare the hypoglycemic effects of four SGLT2 inhibitors, the model simulation results recommend ipragliflozin 50 mg qd in patients with normal renal function and patients with mild renal insufficiency, dapagliflozin 10 mg qd or canagliflozin 100 mg qd in patients with moderate renal insufficiency, and empagliflozin 10 mg qd in patients with severe renal insufficiency to achieve the lowest dose and the maximum therapeutic effect. This study provided a scientific basis for optimizing the dosage regimen in T2DM patients with different degrees of CKD.

## Data Availability

The original contributions presented in the study are included in the article/Supplementary Material, further inquiries can be directed to the corresponding author.
