# Supplementary material for: Physiologically based pharmacokinetic model of sodium-glucose cotransporter 2 inhibitors predicted pharmacokinetics and pharmacodynamics to explore dosage regimen for patients with type 2 diabetes mellitus and renal insufficiency
Source: Front Pharmacol. 2025 Mar 31;16:1520268. doi: 10.3389/fphar.2025.1520268 (PMC11994927; doi:10.3389/fphar.2025.1520268)
Supplement: Supplementary file 1 [file Supplementaryfile1.docx]

***Supplementary Material***

**Exploring reduced efficacy and application of SGLT2 inhibitors in patients with T2DM and renal insufficiency using a physiologically based pharmacokinetic model**

**Author names:** Guimu Guo, Meng Ke, Jianwen Xu, Wanhong Wu, Jiarui Chen, Chengjie Ke, Pinfang Huang, Cuihong Lin

**Corresponding author**: Cuihong Lin

Department of Pharmacy, the First Affiliated Hospital of Fujian Medical University, 20 Cha Zhong M. Rd, Fuzhou 350005, People’s Republic of China.

Department of Pharmacy, National Regional Medical Center, Binhai Campus of the First Affiliated Hospital, Fujian Medical University, Fuzhou, People's Republic of China.

E-mail: Cuihong Lin: [lincuihong1974@sina.com](mailto:lincuihong1974@sina.com)

**Contents**

[**1** **Supplementary Methods** 4](#_Toc173690797)

[1.1 The PD model of patients with T2DM and normal renal function 4](#_Toc173690798)

[1.2 The PD model of patients with T2DM and renal insufficiency 6](#_Toc173690799)

[**2 Supplementary results** 6](#_Toc173690800)

[**3 Supplementary discussions** 7](#_Toc173690801)

[**4 Supplementary tables** 9](#_Toc173690802)

[**Table S1** Demographic characteristics of the simulated population 9](#_Toc173690803)

[**Table S2** Modeling parameters of the UGE model 13](#_Toc173690804)

[**Table S3** The V_max_ and Q_bladder_ values applied to CKD patients in the model 14](#_Toc173690804)

[**5 Supplementary figures** 15](#_Toc173690805)

[**Figure S1** Plasma concentration-time curves of single and multiple oral doses of Dapagliflozin in T2DM patients with normal renal function and renal insufficiency. 16](#_Toc173690807)

[**Figure S2** Cumulative urinary glucose excretion after single and multiple oral doses of Dapagliflozin in T 2 DM patients with normal renal function and renal insufficiency. 18](#_Toc173690808)

[**Figure S3** Plasma concentration-time curves of Canagliflozin after single and multiple oral doses in healthy subjects and T2DM patients with normal renal function. 20](#_Toc173690809)

[**Figure S4** Plasma concentration-time curves of Canagliflozin after a single oral dose in patients with normal renal function, renal insufficiency, and T2DM with renal insufficiency 22](#_Toc173690810)

[**Figure S5** Cumulative urinary glucose excretion after single and multiple oral doses of Canagliflozin in patients with normal renal function, renal insufficiency, and T2DM with renal insufficiency 25](#_Toc173690811)

[**Figure S6** Plasma concentration-time curve of single oral dose of Empagliflozin in healthy subjects and T2DM patients with normal renal function. 26](#_Toc173690812)

[**Figure S7** Plasma concentration-time curve of single oral dose of Empagliflozin in T2DM patients with renal insufficiency. 28](#_Toc173690813)

[**Figure S8** Cumulative urinary glucose excretion after single oral dose of Empagliflozin in patients with renal insufficiency T2DM. 30](#_Toc173690814)

[**Figure S9** Plasma concentration-time curves of single and multiple oral doses of Ipragliflozin in healthy subjects and T2DM patients with normal renal function.. 32](#_Toc173690815)

[**Figure S10** Cumulative urinary glucose excretion after single oral dose of Ipragliflozin in patients with renal insufficiency T2DM. 33](#_Toc173690816)

[Figure S11 Sensitivity analysis results of SGLT2 inhibitor PD model to three parameters of V^SGLT2^_max_, V^SGLT1^_max_ and Q_bladder_. 34](#_Toc173690817)

[**Figure S12** The SGLT2 inhibitors (e.g., dapagliflozin) occupied SGLT2 transporter in T2DM patients with renal insufficiency. 35](#_Toc173690818)

[**Figure S13** Cumulative urinary glucose excretion in T2DM patients with severe renal insufficiency before and after optimization of Empagliflozin PD model. 35](#_Toc173690819)

[**References** 36](#_Toc173690820)

# 1 Supplementary Methods

## 1.1 The PD model of patients with T2DM and normal renal function

The glomerular glucose filtration rate (V^glucose^_GFR_) for each subject was a constant defined as the product of the mean plasma glucose concentration and the glomerular filtration rate (GFR). The mean plasma glucose concentrations in healthy subjects and patients with T2DM were 4.5 and 9.33 mmol/L, respectively (Wang et al., 2022).

$$V_{\mathrm{GFR}}^{\text{glucose}}=GFR\times\text{mean plasma glucose }（S1）$$

The process of glucose flow through the proximal tubules and its excretion into the bladder is a first-order rate process:

$$V_{\text{flux}}^{\text{glucose}}=Q\times\text{Glucos}\text{e}_{\text{conc}}（S2）$$

Where Q is the blood flow and Glucose_conc_ represents the glucose concentration in the S1, S2, and S3 segments.

The total glucose reabsorption rate (GRR) in the renal tubules was the sum of the reabsorption rates of the two sodium-glucose co-transporters, and the absorption rate process was expressed using the Michaelis-Menten equation:

$$GRR=\frac{V_{\text{max}}^{SGLT2}\times\mathrm{Glucos}\text{e}_{\text{S1}}}{K_{m}^{SGLT2}+Glucos\text{e}_{\text{S1}}}+\frac{V_{\text{max}}^{SGLT1}\times\mathrm{Glucos}\text{e}_{\text{S2}}}{K_{m}^{SGLT1}+Glucos\text{e}_{\text{S2}}}（S3）$$

Where GRR is the glucose reabsorption rate; V^SGLT2^_max_ and V^SGLT1^_max_ are the maximum glucose reabsorption rates of SGLT2 and SGLT1, respectively; and K^SGLT2^_m_ and K^SGLT1^_m_ are the affinity constants of glucose for SGLT1 and SGLT2, respectively.

An extended renal tissue model was used to describe the changes in glucose concentration in the S1 segment and urinary glucose excretion over time in patients with T2DM treated with SGLT2 inhibitors. Competitive inhibition of SGLT1 and SGLT2 by SGLT2 inhibitors was described using binding kinetics as shown in the following equation:

$$TO_{\mathrm{SGLT}s,i}=\frac{S_{i}}{S_{\text{free},i}+S_{i}}（S4）$$

Where S_free_ is the concentration of free SGLT1 or SGLT2, and S_i_ represents the concentration of the SGLT2 inhibitor complexed with the SGLT1 or SGLT2 proteins. TO_SGLTs,i_ represents the SGLTs occupancy, and i=1 and 2 represent SGLT1 and SGLT2, respectively.

After the oral administration of SGLT2 inhibitors to patients with T2DM, the drug-specific parameter K_i_ was used to simulate the competitive inhibition of SGLT2 inhibitors on glucose reabsorption in SGLTs:

$$V_{\mathrm{SGLTs}}^{\text{glu}\cos e}=\frac{V_{\text{max}}^{\mathrm{SGLTs}}\times\text{Glucos}\text{e}_{\text{lumen}}}{K_{m}^{\mathrm{SGLTs}}\times（1+\frac{\mathrm{Dru}g_{\mathrm{lumen}}}{Ki^{\mathrm{SGLTs}}}）+\text{Glucos}\text{e}_{\text{lumen}}} （S5）$$

Where V_SGLTs_^glucose^ is the SGLT-mediated glucose reabsorption concentration after using the SLGT2 inhibitor, Drug_lumen_ represents the concentration of the SGLTs inhibitor in the corresponding lumen, and K_i,SGLTs_ is the affinity constant of the SGLT2 inhibitor for SGLT1 or SGLT2.

The SGLTs-mediated glucose reabsorption capacity of the proximal renal tubules increases as the glomerular glucose filtration rate increases until the maximum glucose transport capacity (TmG) is reached. The sum of glucose reabsorption of the two glucose co-transporters after SGLT2-inhibitor treatment in patients with T2DM is shown below:

$$T\text{mG}=V_{SGLT1}^{\text{glu}\cos e}+V_{SGLT2}^{\text{glu}\cos e}（S6）$$

An additional process was added to the MoBi software to represent glucose accumulation in the bladders of patients with T2DM taking SGLT2 inhibitors.

$$V_{\mathrm{bladder}}^{\text{glucose}}=（\mathrm{GFR}\times\text{mean plasma glucose-TmG}+V_{\text{S1}}^{\text{glucose}}+V_{\text{S2}}^{\text{glucose}}）\times MW/Q_{\mathrm{bladder}}（S7）$$

Where MW is the relative molecular weight of glucose, Q_bladder_ is the bladder flow rate, and V_S1_^glucose^ and V_S2_^glucose^ represent the glucose concentration in S1 and S2 lumen, respectively.

The maximal glucose reabsorption capacities of SGLT1 and SGLT2 in patients with T2DM compared to healthy subjects increased by 54% and 28%, respectively. Yakovleva et al. reported V^SGLT2^_max_ and V^SGLT1^_max_ values of an UGE model to represent the maximum glucose reabsorption capacity of SGLT1 and SGLT2 in patients with T2DM and healthy subjects (28.6 vs 18.53 and 111.4 vs 87.07 mmol/h), respectively (Yakovleva et al., 2019). The SGLT2-inhibitor PD model was validated using the observed 24-h urinary glucose excretion values in healthy subjects and patients with T2DM.

## 1.2 The PD model of patients with T2DM and renal insufficiency

Using the parameter identification block in the model, we performed a sensitivity analysis of the SGLT2-inhibitor PD model, in which V^SGLT2^_max_, V^SGLT1^_max_, and Q_bladder_ significantly affected urinary glucose excretion in patients with T2DM and renal insufficiency (**Figure S11**). The observed values of urinary glucose excretion were combined with the results of sensitivity analysis. In patients with mild, moderate and severe renal insufficiency, the V^SGLT1^_max_ values were set to 42.9, 24.31 and 14.3 mmol/h, the V^SGLT2^_max_ values were set to 93, 44.56 and 11.14 mmol/h, and the Q_bladder_ values were set at 0.72, 0.52, 0.20 L/h, respectively.

# 2 Supplementary results

The maximum reabsorption rate of the SGLTs were used to quantitatively assess renal glucose reabsorption capacity. The sensitivity analysis results of the PD model in patients with T2DM and renal insufficiency indicated that the three physiological parameters V^SGLT1^_max_, V^SGLT2^_max_, and Q_bladder_ significantly affected the urinary glucose excretion of patients using the parameter values provided in the PK-Sim software (**Figure S11**). The dapagliflozin and empagliflozin PD model fitting results showed that when the V^SGLT1^_max_ value increased by 50% and the V^SGLT2^_max_ value decreased by 16.5% in patients with T2DM and mild renal insufficiency compared to patients with T2DM and normal renal function, the fold error of the 24-h urinary glucose excretion between the model-predicted values and the clinically measured values was the smallest.

Nakamura et al. used nephrectomized rats as a chronic renal failure model to detect the activity and expression level of SGLTs transporters, and the results showed that the reabsorption capacity of SGLT1 and SGLT2 in chronic-renal-failure rats was significantly decreased, with SGLT1 decreasing by 56.7% and SGLT2 decreasing by 47.8% (Nakamura et al., 2004). Application the PD model of patients with T2DM and moderate or severe renal insufficiency combined with the 24-h UGE observed values and the parameters estimated by PK-Sim software optimized the three physiological parameters of V^SGLT1^_max_, V^SGLT2^_max_, and Q_bladder_. In patients with moderate and severe renal insufficiency, when the three parameters V^SGLT1^_max_, V^SGLT2^_max_, and Q_bladder_ were decreased by 15%, 50%, and 60%; and 90%, 27%, and 72%, respectively, the fold error of the 24-h UGE between the model-predicted values and the observed values was minimal.

The PBPK model simulated the change in SGLT2 inhibitor occupancy of the SGLT2 transporter with time, as shown in **Figure S12**. In patients with T2DM and normal renal function or mild, moderate, or severe renal insufficiency, the maximum SGLT2 inhibitor occupancy values of the SGLT2 transporter were 98.35%, 97.41%, 95.82%, and 90.59%, respectively. Although the maximum occupancy of the SGLT2 transporters by SGLT2 inhibitors decreased as renal damage became aggravated, the maximum occupancy remained above 90%. This result partially explains why SGLT2 inhibitors maintained their effectiveness in patients with renal insufficiency despite UGE decreasing as the GFR decreased. In addition, overexpression of SGLTs at other sites, such as the intestines, is also a compensatory mechanism for urinary glucose excretion after renal failure.

Based on the results of the PD model, this study found that the decrease in the glucose reabsorption capacity of SGLT1 and SGLT2 transporters in renal tubules was another important reason for the reduced efficacy of SGLT2 inhibitors. Additionally, this research provides new ideas and a theoretical basis for future research on SGLT2 inhibitors in patients with renal insufficiency.

# 3 Supplementary discussions

Sodium-glucose co-transporter (SGLT) play a crucial role in glucose reabsorption. SGLT1 is primarily located on the luminal side of epithelial cells in the small intestine and on the luminal side of the third segment (S-3) of the proximal renal tubule, whereas SGLT2 is primarily located on the luminal side of the first (S-1) and second (S-2) segments of the proximal renal tubule (L. H. Chen & Leung, 2013; Vrhovac et al., 2015). SGLT2 inhibitors function to lower blood sugar by inhibiting renal SGLT2, leading to increased urinary glucose excretion (UGE). In addition to inhibiting renal SGLT2, canagliflozin also inhibits intestinal SGLT1. However, to effectively inhibit intestinal SGLT1, canagliflozin must maintain a high concentration level within the intestinal lumen, which requires high intestinal permeability. Mori K, et al., using a PBPK model, predicted the concentrations and effects of canagliflozin in the human intestinal tract and renal tubules. Their simulation results showed that the concentration of canagliflozin in the intestinal lumen is significantly higher than that of dapagliflozin (Mori et al., 2016). As there are no current literature reports on the specific intestinal permeability value of canagliflozin, this study combines the results from Mori K and the actual data set of healthy subjects treated with canagliflozin by Devineni D, et al. (Devineni, Murphy, et al., 2015), and uses the parameter optimization module in PKsim software to optimize the specific intestinal permeability value of canagliflozin to 0.02196 cm/min.

An interesting finding from this study is that the reabsorption capacity of the SGLT1 transporter increased in patients with T2DM and mild renal insufficiency but decreased in patients with T2DM and moderate or severe renal insufficiency. However, the reabsorption capacity of the SGLT2 transporter decreased across all stages of CKD. Further, Kim et al. showed that the Na-K-2Cl cotransporter (NKCC2) and Na-Cl cotransporter (NCC) also appeared in the kidney tissues across early and late stages, compared with a control group (rats with normal renal function), the NKCC2 and NCC expression levels in kidney tissue increased by 477% and 222%, respectively, in early CKD stages in 5/6 nephrectomized rats and decreased by 55.4% and 30.8%, respectively, in later stages, and this research results indirectly supported our findings (Kim et al., 2010). The pathophysiological mechanism behind the increased reabsorption capacity of the SGLT1 transporter in patients with T2DM and mild renal insufficiency involves diabetic hyperfiltration, which enhances glucose and sodium reabsorption in renal proximal tubules. This process leads to compensatory upregulation of renal SGLTs, and tubular growth (Vallon & Thomson, 2020). Furthermore, hyperglycemia stimulates the transcription level of serum and glucocorticoid-induced protein kinase-1 (SGK-1), as well as the expression of Na/H exchange protein (NHE-3), SGLT1, and other transporters (Yang & Xu, 2022). In patients with T2DM and moderate or severe renal insufficiency, the pathophysiological mechanism affecting SGLT transport protein reabsorption involves the accumulation of uremic toxins such as indole sulfate, and hippuric acid in the circulation and tissues. These toxins promote renal fibrosis and endothelial dysfunction, resulting in structural and functional damage to the kidneys (Lim et al., 2021).

The SGLT1 transporter plays an important role in glucose reabsorption in patients with T2DM and renal insufficiency. We found that in the severe renal insufficiency PD model, when both V^SGLT1^_max_ and V^SGLT2^_max_ decrease by 90%, the 24 h UGE of patients is underestimated. However, when V^SGLT1^_max_ decreased by 60% and V^SGLT2^_max_ by 90%, the predicted UGE curve was more consistent with the clinically measured data points (**Figure S13**). SGLT2 inhibitors increase glucose availability to SGLT1 transporters located downstream in the proximal tubules of the kidney, leading to a compensatory increase in SGLT1-mediated tubular glucose reabsorption (Wang et al., 2022).

**4 Supplementary tables**

**Table S1** Demographic characteristics of the simulated population

| Drug | Race | Renal function | Dosage regimen | Number | Years | Weight | BMI (kg/m^2^) | References |
| --- | --- | --- | --- | --- | --- | --- | --- | --- |
| Dapagliflozin | Japanese T2DM | normal | Po 10, 20mg SAD | 32 | 23(20–45) | 64.3 | 18–25 | \| (Kasichayanula et al., 2011) \| \| --- \| \| |
|  |  |  | Po 10, 20mg qd, for 14 consecutive days | 36 | 64(20–70) | 61 | <32 |  |
|  | Caucasian T2DM | normal | \| Po 50mg SAD \| \| --- \| \| \| \| | 8 | 43(25–63) | 77.9(62.2–96.0) | 27.8(22.7–31.2) | \| (Kasichayanula et al., 2014) \| \| --- \| \| \| \| |
|  |  | mild |  | 8 | 66(55–75) | 73.9(58.8–90.6) | 29.3(21.1–34.3) |  |
|  |  | moderate |  | 8 | 72(62–76) | 77.4(51.8–101.2) | 27.9(20.2–32.7) |  |
|  |  | severe |  | 4 | 66(59–73) | 78.4(72.3–80.9) | 27.2(26.2–28.7) |  |
| \| Canagliflozin \| \| --- \| \| \| \| \| \| \| | Japanese T2DM | normal | Po 25、100、200、400mg qd, for 16 consecutive days | 61 | 54.3 (8.8) | 70.84 (12.38) | 25.56 (3.39) | (Iijima et al., 2015) |
|  | Japanese T2DM | normal | Po 100、200 SAD | 12 | 57.8 (9.7) | 75.33(10.38) | 25.75(3.32) | (Inagaki et al., 2014) |
|  |  | moderate |  | 12 | 63.7(10.2) | 74.85(7.01) | 26.33(1.98) |  |
|  | Chinese HS | normal | Po 100、300 SAD | 15 | 29.5 (4.90) | 63.0 (7 .32) | 22.0 (2.08) | (X. Chen et al., 2015) |
|  | Caucasian HS | normal | Po 300mg SAD | 9 | 39.2 (9.83) | 78.2(8.56) | 25.2 (2.45) | (Devineni, Murphy, et al., 2015) |
|  | Caucasian | normal | \| Po 200mg SAD \| \| --- \| \| \| \| | 8 | 52.0 (42–65) | 92.5 (82.4–105.9) | 31.2 (27–33) | \| (Devineni, Curtin, et al., 2015) \| \| --- \| \| \| \| |
|  |  | mild |  | 8 | 59.8 (31–75) | 70.1 (61.0–85.4) | 25.7 (22–28) |  |
|  |  | moderate |  | 8 | 62.6 (29–77) | 77 .7 (54.0–97.3) | 28.1 (20–36) |  |
|  |  | severe |  | 8 | 61.3 (45–77) | 72.7 (51.5–88.1) | 24.8 (20–28) |  |
| Ipragliflozin | Caucasian HS | normal | Po 5mg、30mg、100mg qd, for 14 consecutive days | 24 | 38.2(24–46) | - | 24.4(21.9-26.8) | (Veltkamp et al., 2011) |
|  | Caucasian HS | normal | Po 100mg SAD | 8 | 55.6(46.0–63.0) | 74.2 (60.8–80.1) | 26.2 (21.4–32.0) | (Zhang et al., 2013) |
|  | Japanese T2DM | mild | \| Po 50 mg SAD \| \| --- \| \| \| | 8 | 55±12 | - | 27.3±2.1 | \| (Ferrannini et al., 2013) \| \| --- \| \| \| |
|  |  | moderate |  | 9 | 61±11 | - | 25.1±3.8 |  |
|  |  | severe |  | 9 | 64±10 | - | 24.0±2.9 |  |
| Empagliflozin | Caucasian HS | normal | Po 50 mg SAD | 16 | 32.5 (22– 48) | 81.5 (65–100) | 25.5 (22.0 –28.9) | (Friedrich et al., 2013) |
|  | Caucasian HS | normal | Po 50 mg SAD | 5 | 18–50 |  | 18.5–29.9 | (Seman et al., 2013) |
|  | Caucasian HS | normal | Po 50 mg SAD | 16 | 38.5 (20–49) | 78.0 (60–98) | 24.2  (20.8–28.1) | (Brand et al., 2012) |
|  | Chinese T2DM | normal | Po 25 mg SAD | 24 | 53.5 (32–68) | 68.0 (50–86) | 25.5 (19.2–32.4) | (Zhao et al., 2015) |
|  | Japanese T2DM | normal | \| Po 25 mg SAD \| \| --- \| \| \| \| | 8 | 67 .0 (49–75) | 63.35 (45.1–77 .0) | 24.50 (18.3–28.7) | \| (Sarashina et al., 2014) \| \| --- \| \| \| \| |
|  |  | mild |  | 8 |  |  |  |  |
|  |  | moderate |  | 8 |  |  |  |  |
|  |  | severe |  | 8 |  |  |  |  |
|  | Caucasian | normal | \| Po 50 mg SAD \| \| --- \| \| \| \| | 8 | 56 (34–74) | 78.30 (60.5–103.0) | 27.65 (19.6–34.0) | \| (Macha et al., 2014) \| \| --- \| \| \| \| |
|  |  | mild |  | 9 |  |  |  |  |
|  |  | moderate |  | 7 |  |  |  |  |
|  |  | severe |  | 8 |  |  |  |  |

Po represented oral; qd represented once a day; SAD represented administered single dose；T2DM represented type 2 diabetic patient; HS represented health volunteer; GFR > 90 ml/min in healthy subjects and T2DM patients with normal renal function; 60-90 ml/min in T2DM patients with mild renal insufficiency; 30-60 ml/min in T2DM patients with moderate renal insufficiency; < 30 ml/min in T2DM patients with severe renal insufficiency.

**Table S2** Modeling parameters of the UGE model

| Parameter | Description | Values used in the model |
| --- | --- | --- |
| C_glucose_^HS^ | Average plasma glucose concentration in healthy subjects | 5.5mM (Yakovleva et al., 2019) |
| C_glucose_^T2DM^ | Average plasma glucose concentration in T2DM patients | 9.33mM (Yakovleva et al., 2019) |
| V_lumen_^S1^ | S1 segment volume | 0.045L (Yakovleva et al., 2019) |
| V_lumen_^S2^ | S2 segment volume | 0.019L (Yakovleva et al., 2019) |
| V_bladder_ | Bladder volume | 0.2L (Yakovleva et al., 2019) |
| Q_lumen S1/S2_ | Flow rate in segment S1/S2 | 2.7L/h (Yakovleva et al., 2019) |
| Q_bladder_ | Flow rate in bladder | 0.72L/h (Yakovleva et al., 2019) |
| Q_urine_ | Flow rate in urine | 0.055L/h (Yakovleva et al., 2019) |
| K_m_^SGLT1^ | Michaelis-Menten constant of glucose for SGLT1 | 0.5mM (Yakovleva et al., 2019) |
| K_m_^SGLT2^ | Michaelis-Menten constant of glucose for SGLT2 | 4mM (Yakovleva et al., 2019) |
| V_max_^SGLT2-HS^ | SGLT2 maximal glucose reabsorption rate in healthy subject | 87.07mmol/h (Yakovleva et al., 2019) |
| V_max_^SGLT2-T2DM^ | SGLT2 maximal glucose reabsorption rate in T2DM patient | 111.4mmol/h (Yakovleva et al., 2019) |
| V_max_^SGLT1-HS^ | SGLT1 maximal glucose reabsorption rate in healthy subject | 18.53mmol/h (Yakovleva et al., 2019) |
| V_max_^SGLT1-T2DM^ | SGLT1 maximal glucose reabsorption rate in T2DM patient | 28.6mmol/h (Yakovleva et al., 2019) |

HS indicated healthy subjects; T2DM indicated patients with type 2 diabetes; SGLT1/2 indicated sodium-glucose cotransporter 1/2

**Table S3** The V_max_ and Q_bladder_ values applied to CKD patients in the model

| Renal function | V_max_^SGLT2^(mmol/h) | V_max_^SGLT1^(mmol/h) | Q_bladder_(L/h) |
| --- | --- | --- | --- |
| normal | 111.4 | 28.6 | 0.72 |
| mild | 93 | 42.9 | 0.72 |
| moderate | 44.56 | 24.31 | 0.52 |
| severe | 11.14 | 14.3 | 0.2 |

V_max_ indicated the SGLT2 maximal glucose reabsorption rate in CKD patient; Q_bladder_ indicated the flow rate in bladder.

**5 Supplementary figures**

A

B


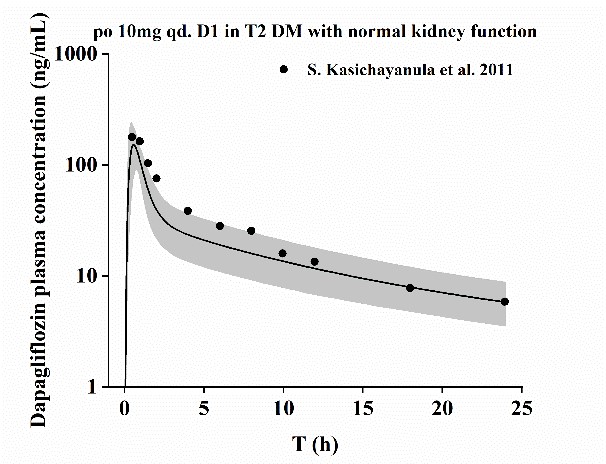

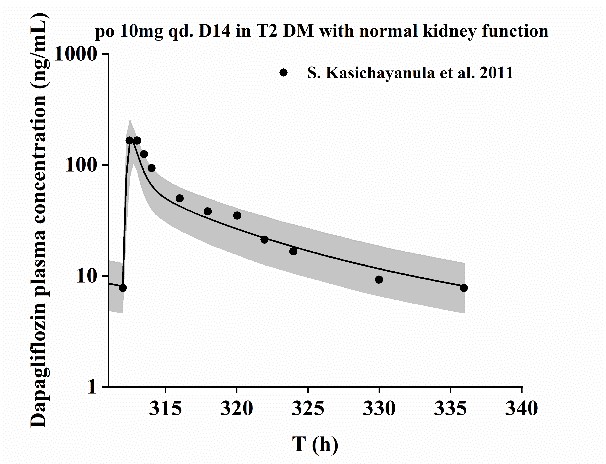


D

C


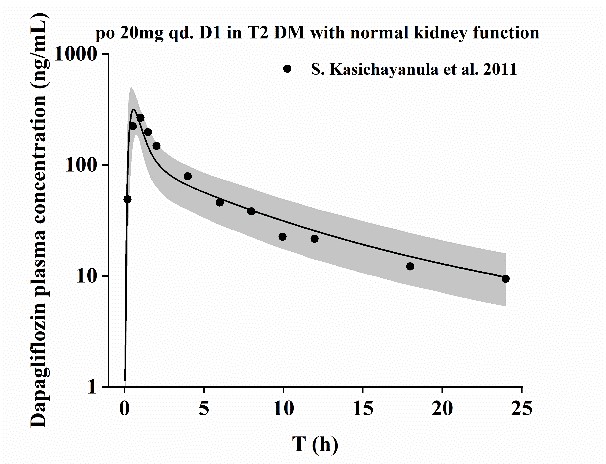

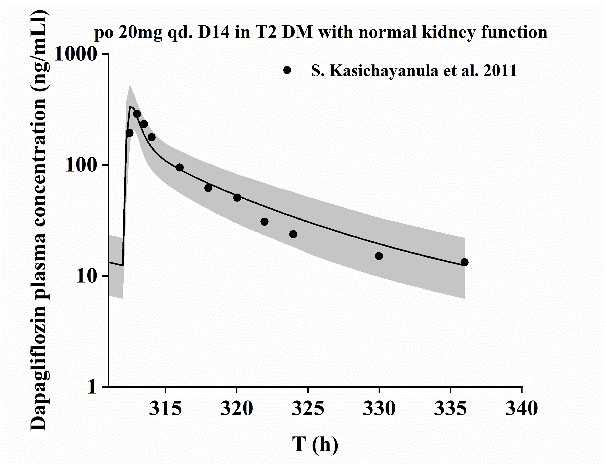


F

E


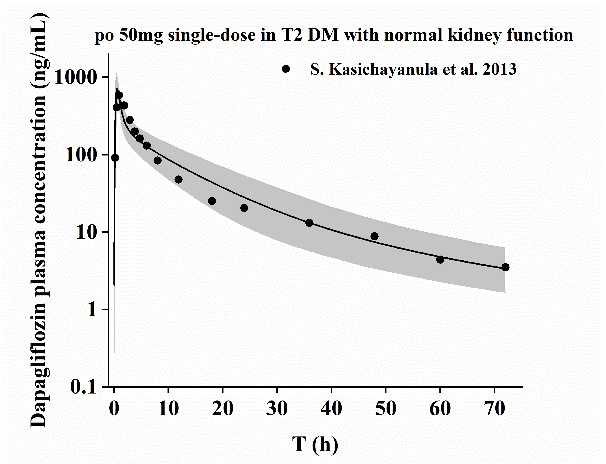

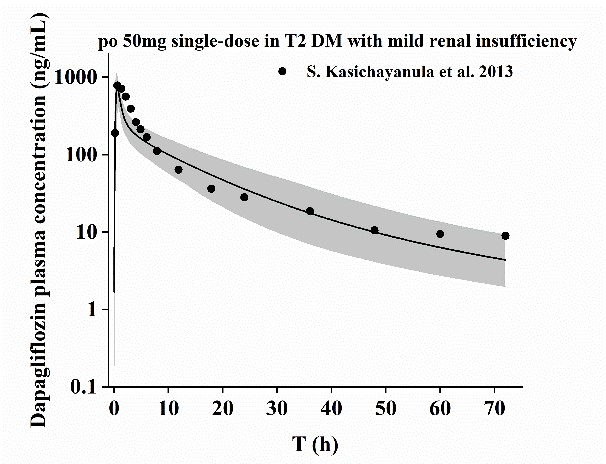


H

G


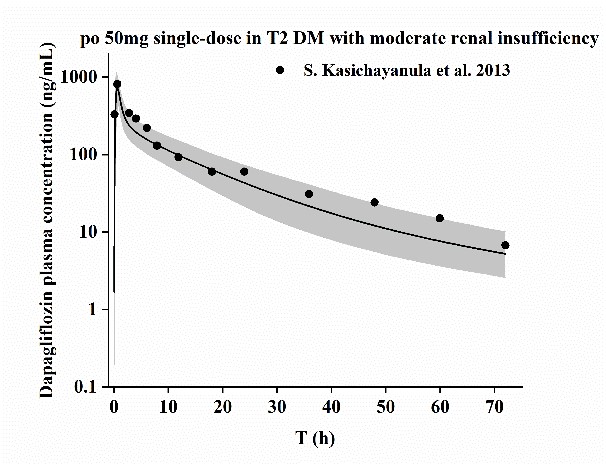

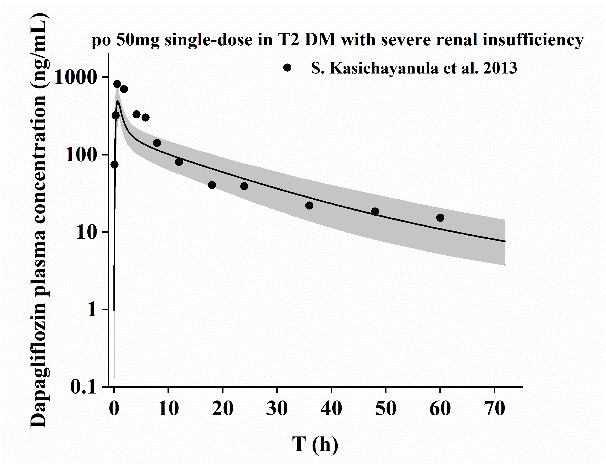


**Figure S1** Plasma concentration-time curves of single and multiple oral doses of Dapagliflozin in T2DM patients with normal renal function and renal insufficiency. Po: oral administration; qd.: once a day; T2DM: type 2 diabetic patients; D1: day 1; D14: day 14; grey shading was 90% confidence interval; black dots were measured values. (A-D) Clinical data of patients with normal renal function were obtained from S. Kasichayanula et al., 2011 (Kasichayanula et al., 2011); (E-H) Clinical data of patients with renal insufficiency were obtained from Kasichayanula et al., 2014 (Kasichayanula et al., 2014).

A

B


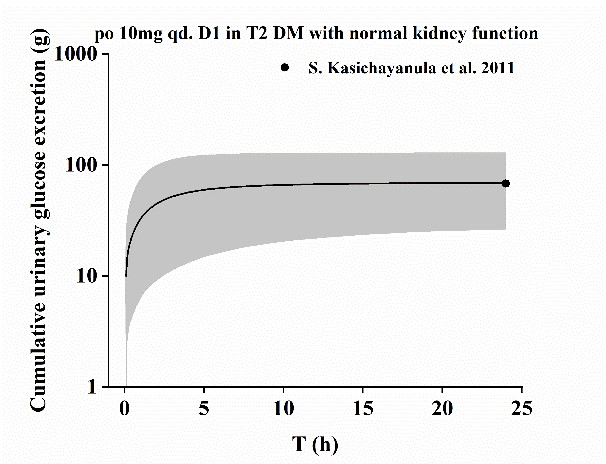

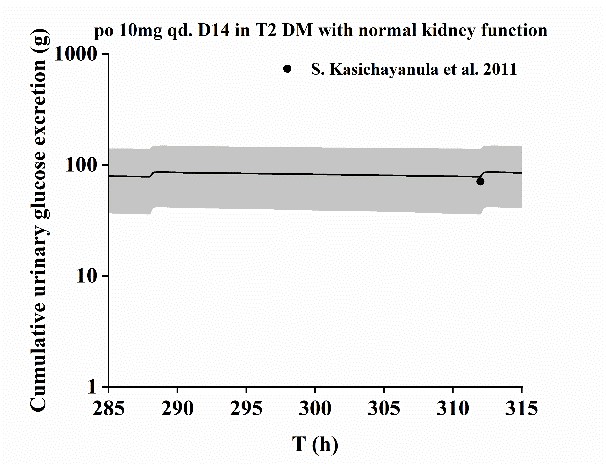


D

C


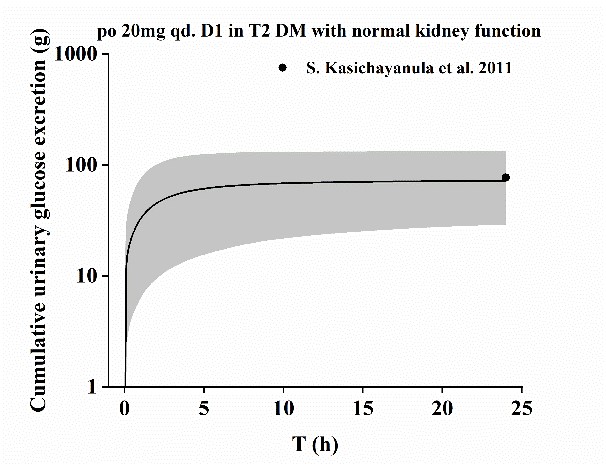

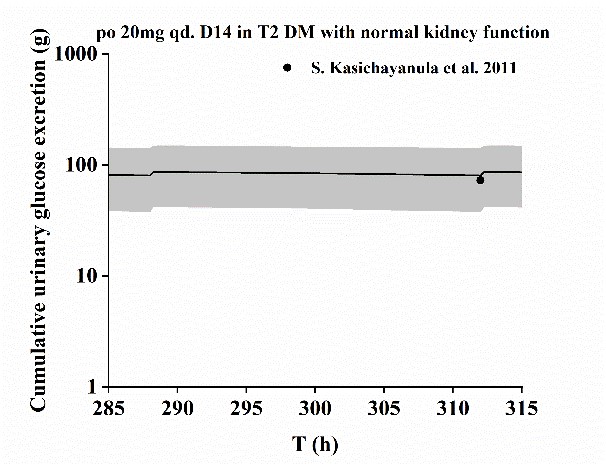


F

E


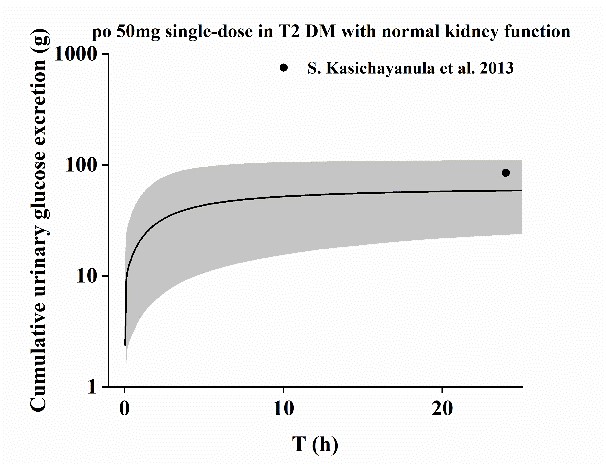

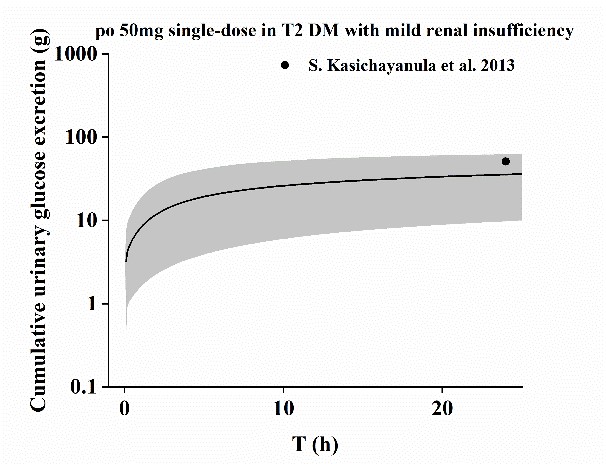


H

G


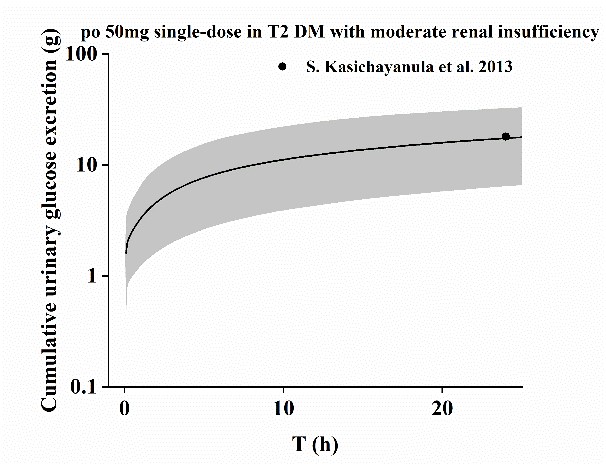

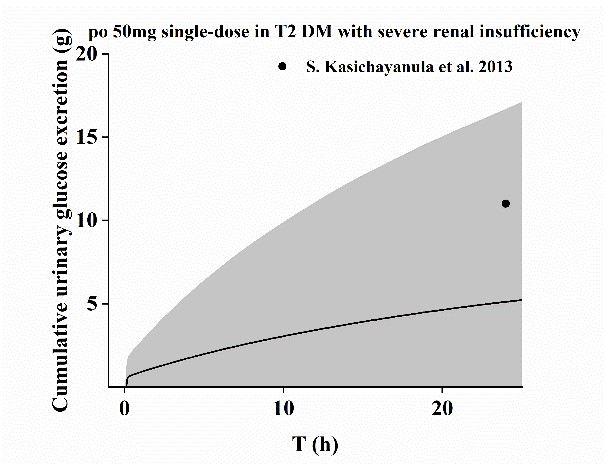


**Figure S2** Cumulative urinary glucose excretion after single and multiple oral doses of Dapagliflozin in T2 DM patients with normal renal function and renal insufficiency. Po: oral administration; qd.: once a day; T2DM: patients with type 2 diabetes; D1: day 1; D14: day 14; grey shading was 90% confidence interval; black dots were measured values. (A-D) Clinical data of patients with normal renal function were obtained from Kasichayanula et al., 2011 (Kasichayanula et al., 2011). (E-H) Clinical data of patients with renal insufficiency were obtained from Kasichayanula et al., 2014 (Kasichayanula et al., 2014).

A

B


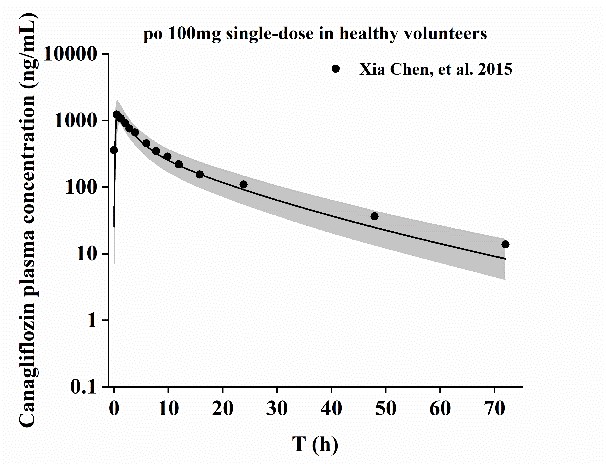

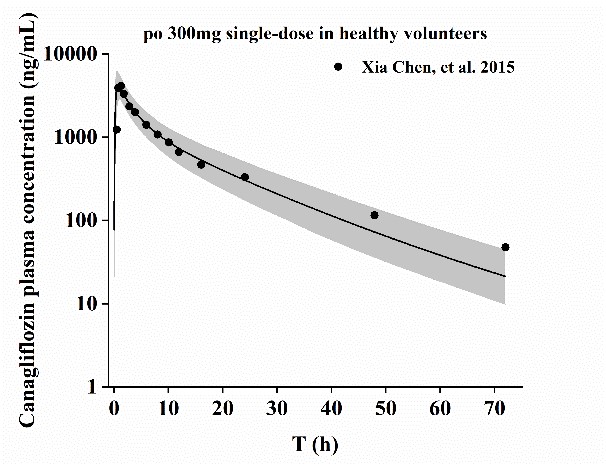


D

C


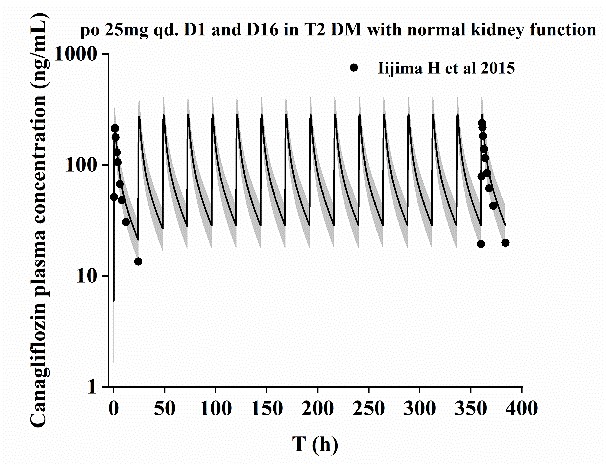

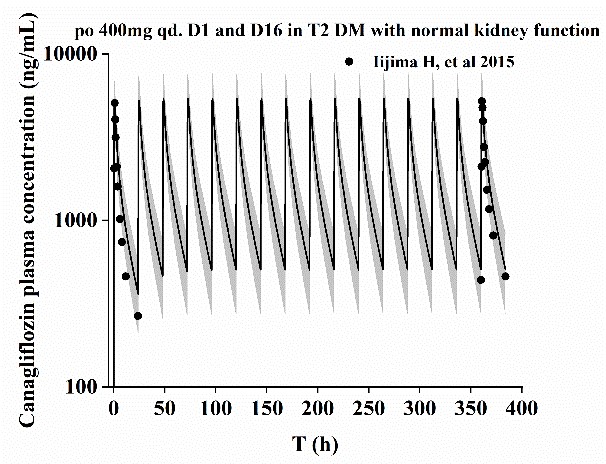


F

E


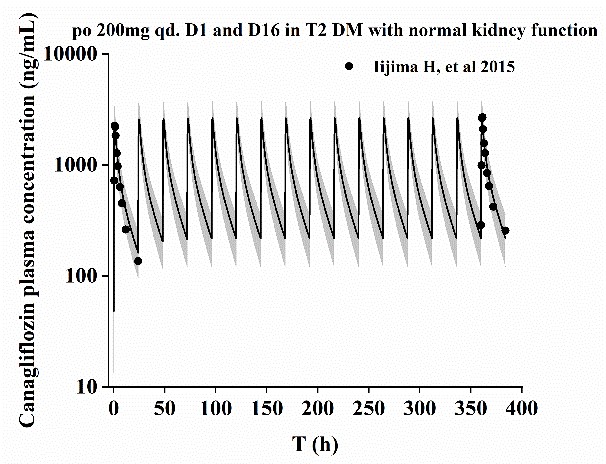

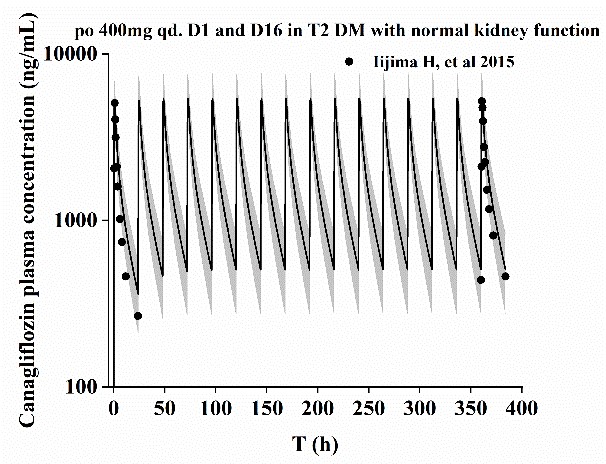


G


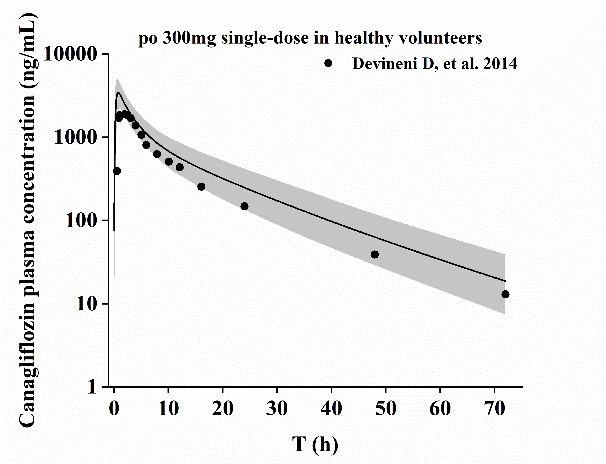


**Figure S3** Plasma concentration-time curves of Canagliflozin after single and multiple oral doses in healthy subjects and T2DM patients with normal renal function. Po: oral administration; qd.: once a day; T2DM: type 2 diabetic patients; D1: day 1; D16: day 16; grey shading was 90% confidence interval; black dots were measured values. (A, B) Clinical data of healthy subjects were obtained from Chen et al., 2015 (X. Chen et al., 2015); (C-F) Clinical data of patients with normal renal function were obtained from Iijima et al., 2015 (Iijima et al., 2015).

A

B


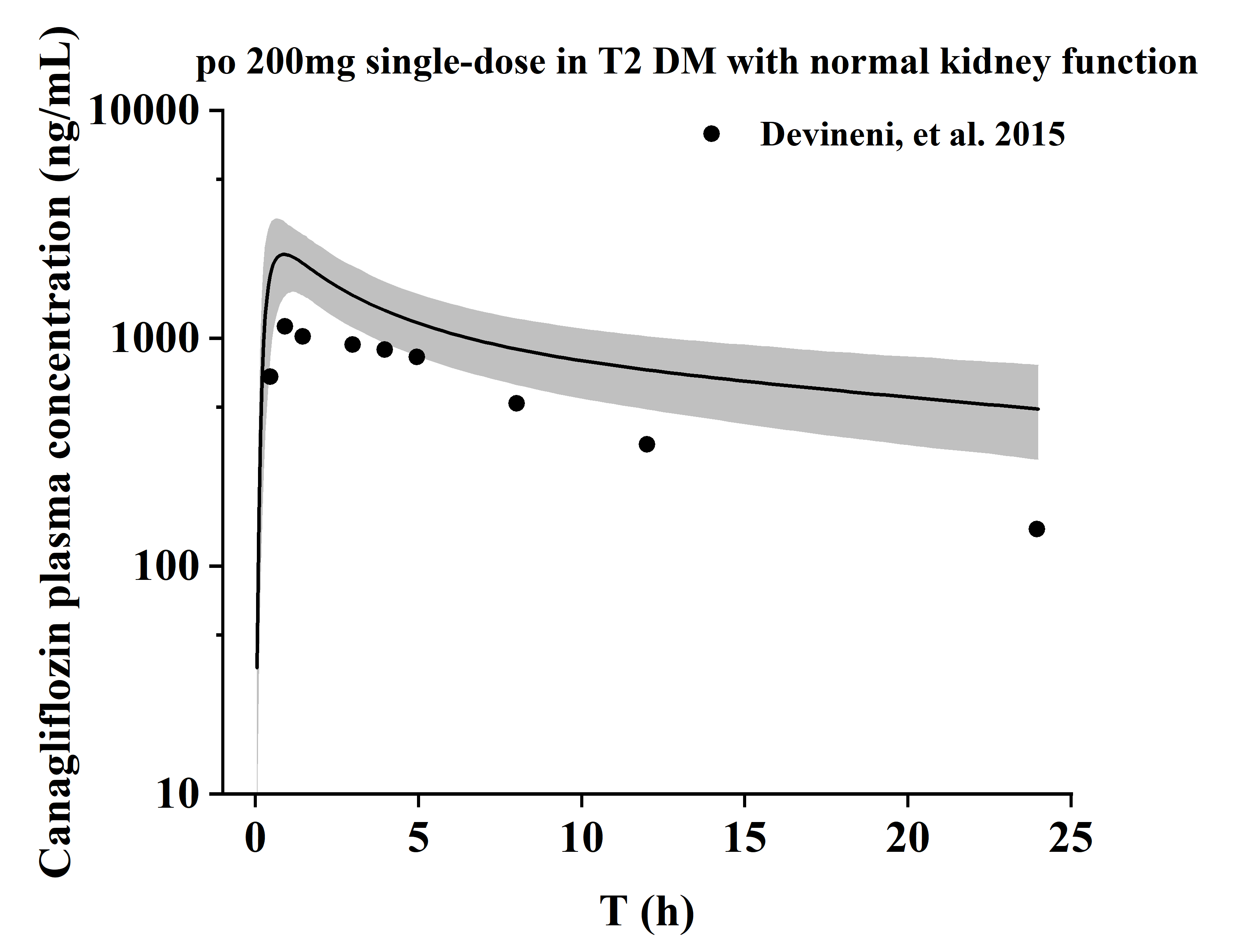

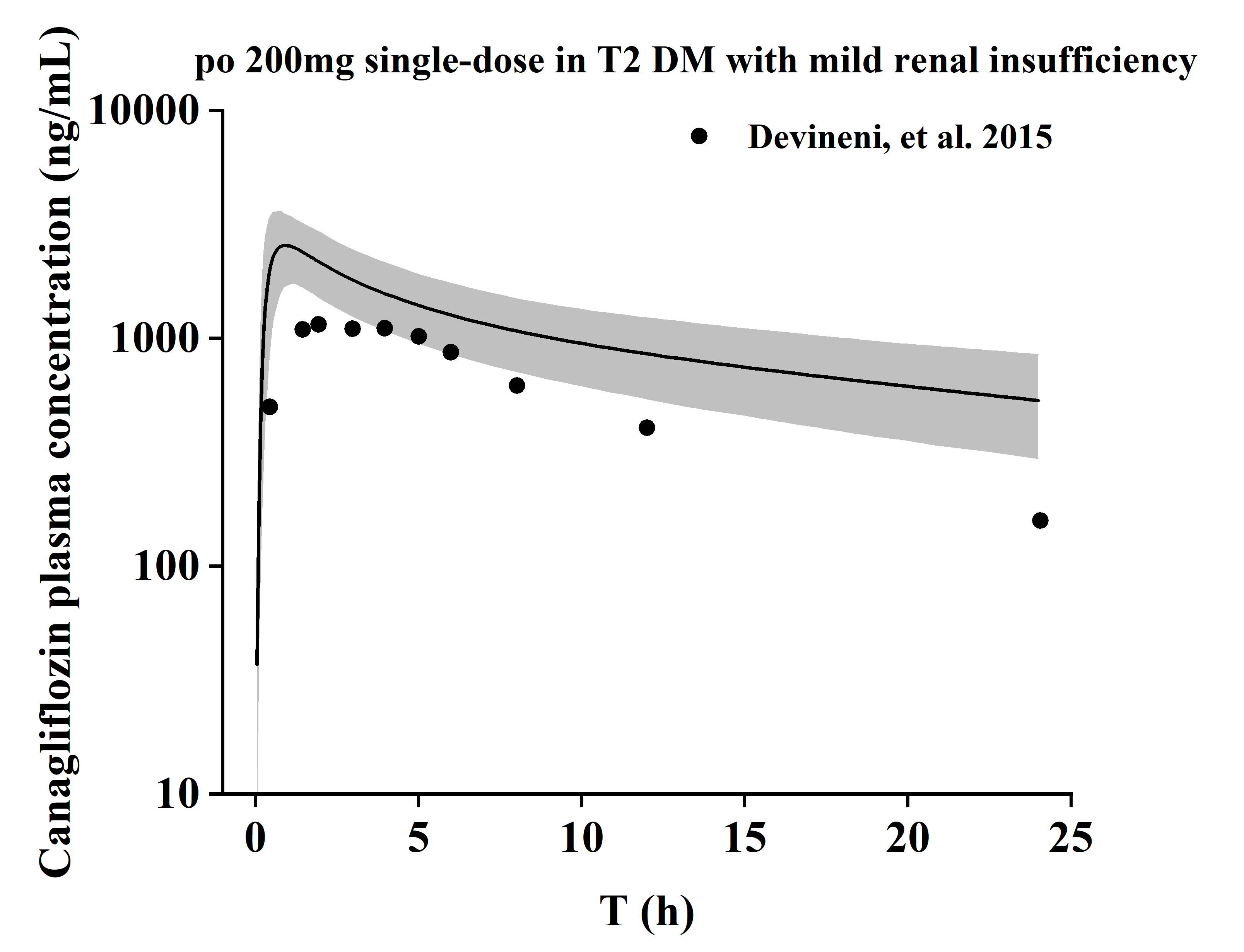


D

C


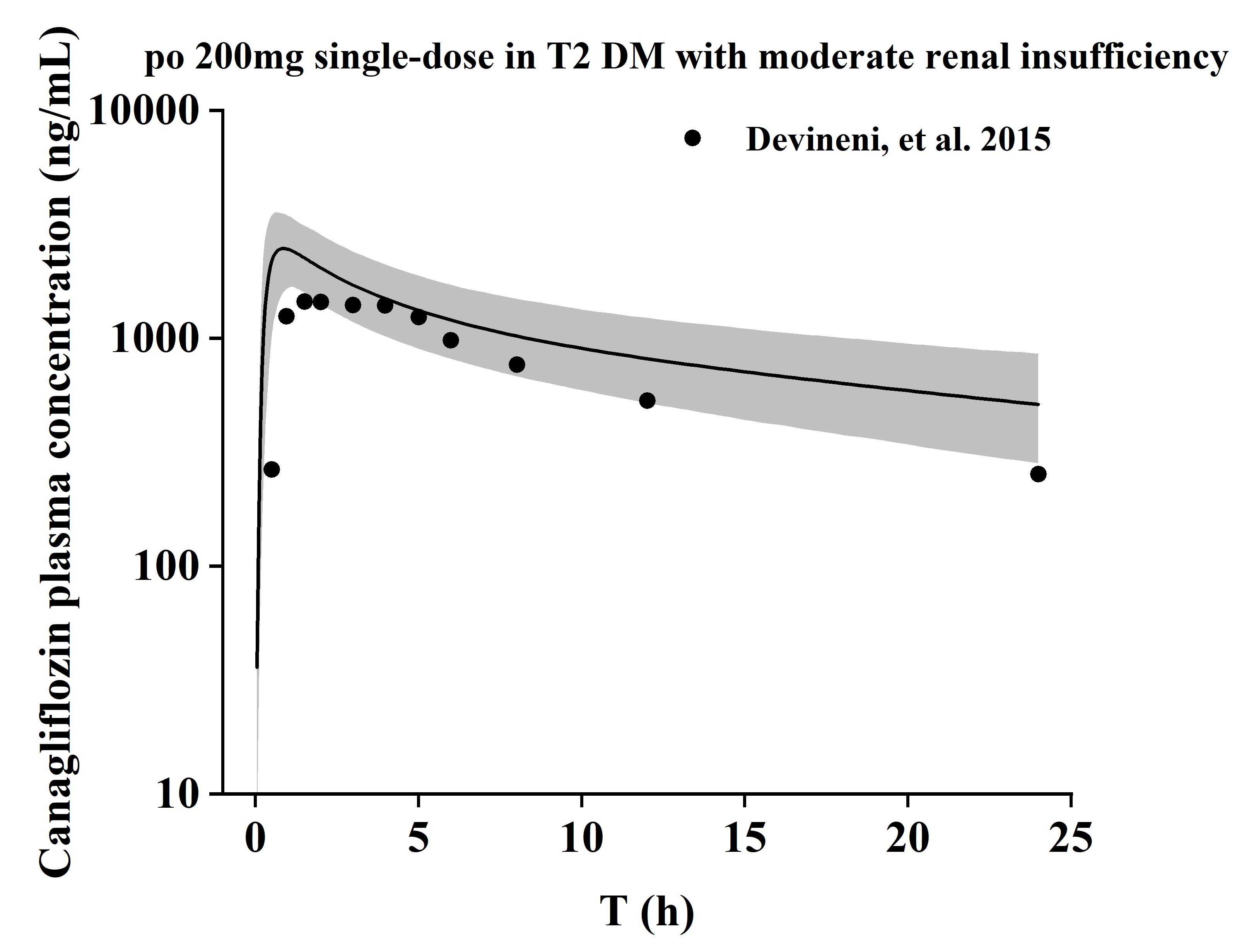

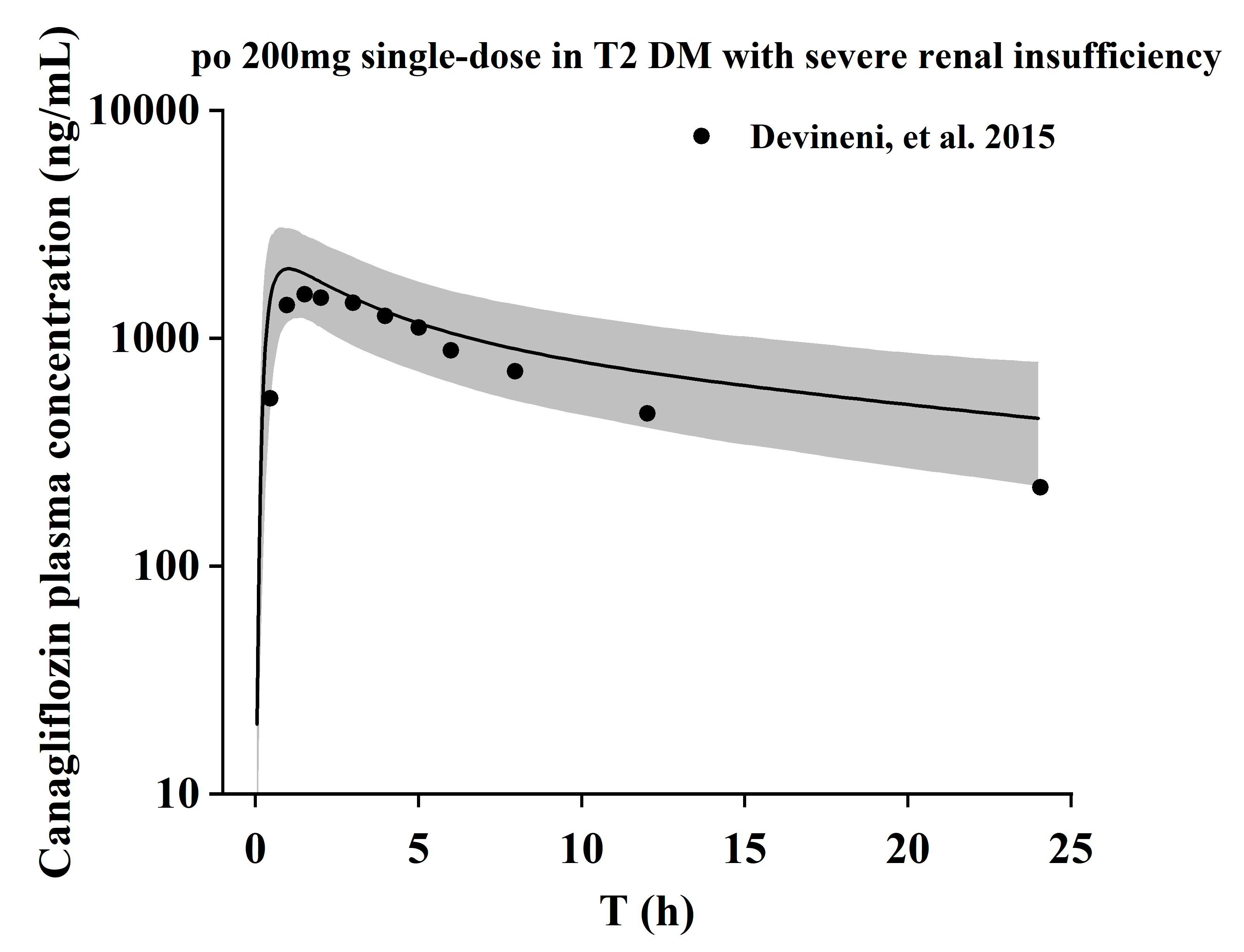


F

E


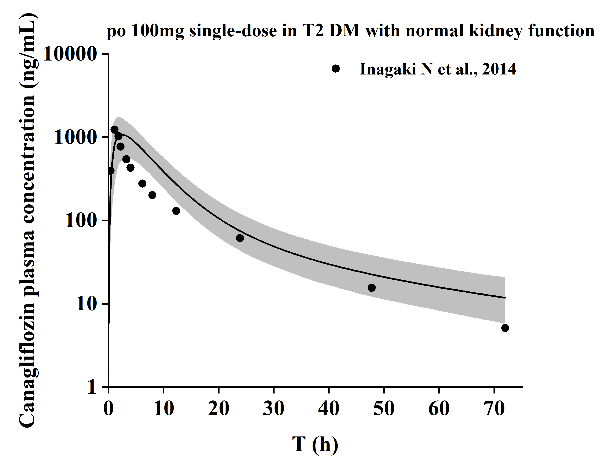

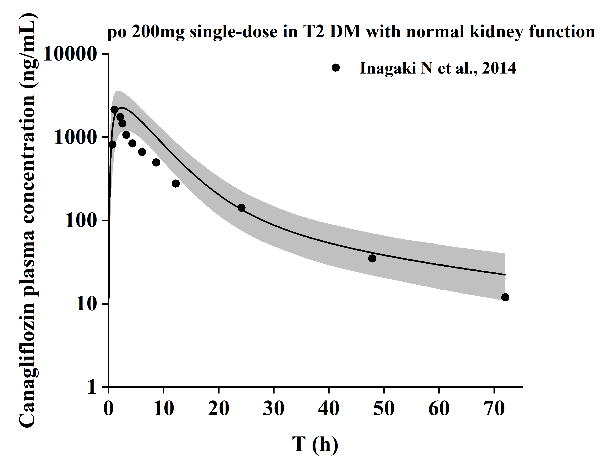


H

G


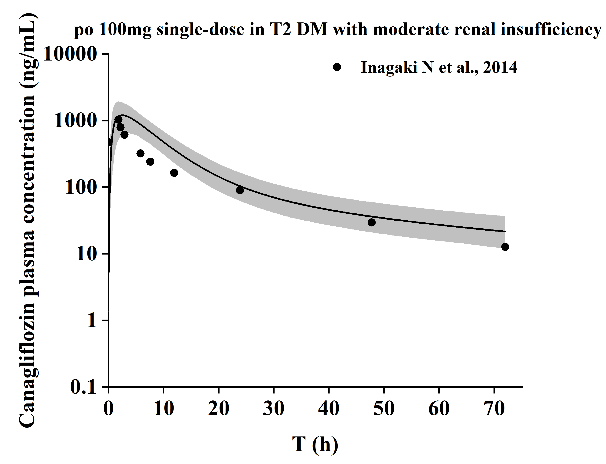

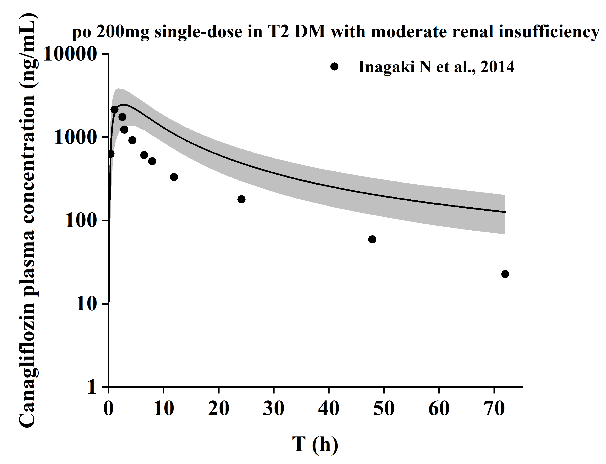


**Figure S4** Plasma concentration-time curves of Canagliflozin after a single oral dose in patients with normal renal function, renal insufficiency, and T2DM with renal insufficiency. Po: oral administration; T2DM: type 2 diabetes mellitus; grey shading was 90% confidence interval; black dots were measured values. (A-D) Clinical data of patients with renal insufficiency were obtained from Devineni et al., 2015 (Devineni, Curtin, et al., 2015). (E-H) Clinical data of patients with renal insufficiency were obtained from Inagaki N et al., 2014 (Inagaki et al., 2014).

A

B


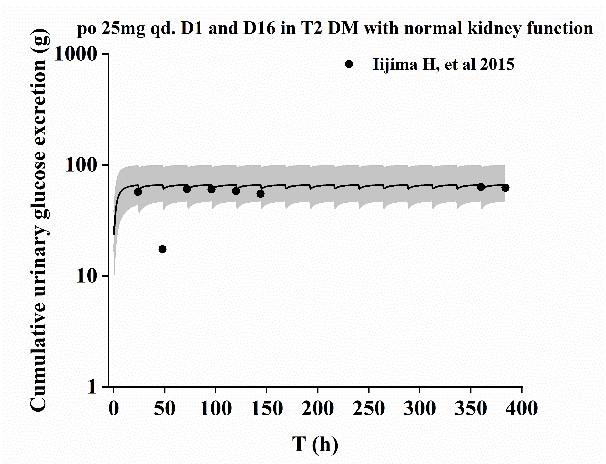

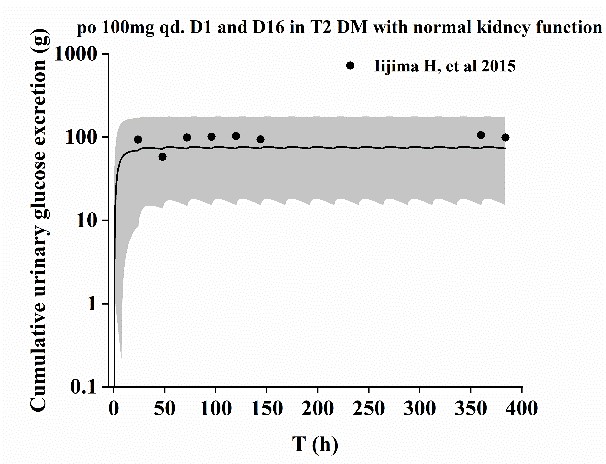


D

C


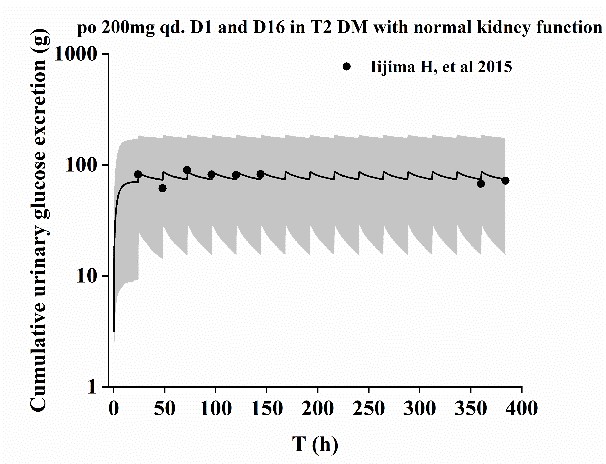

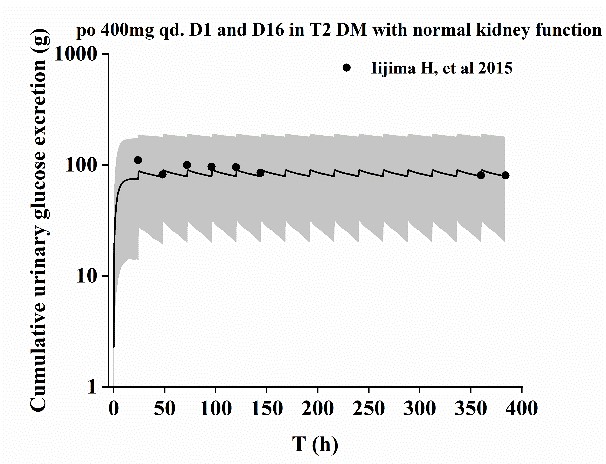


F

E


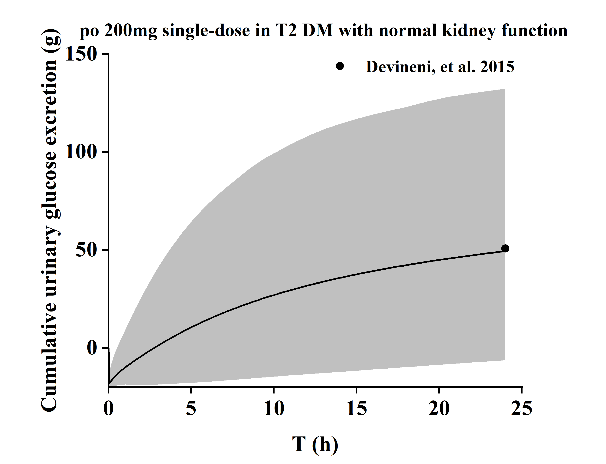

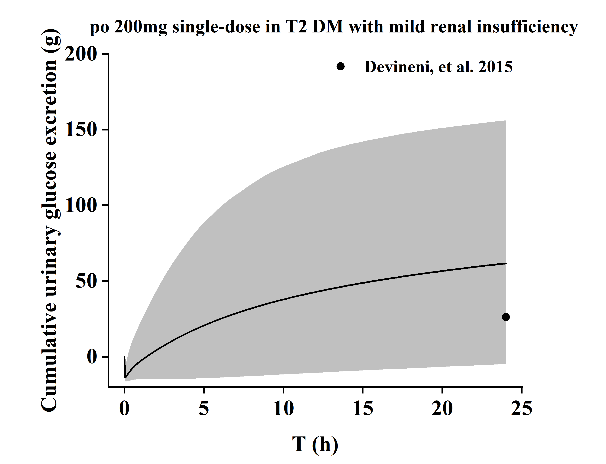


H

G


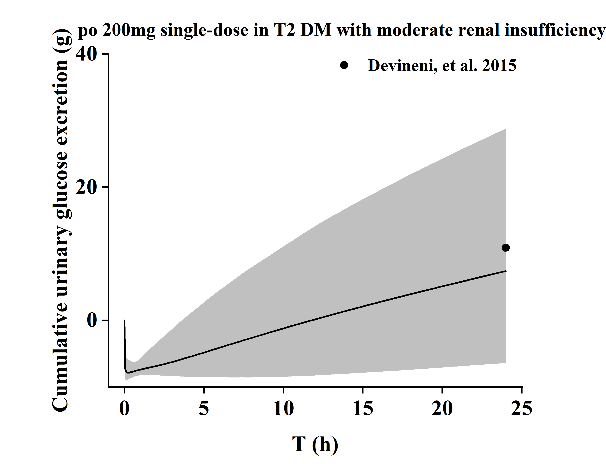

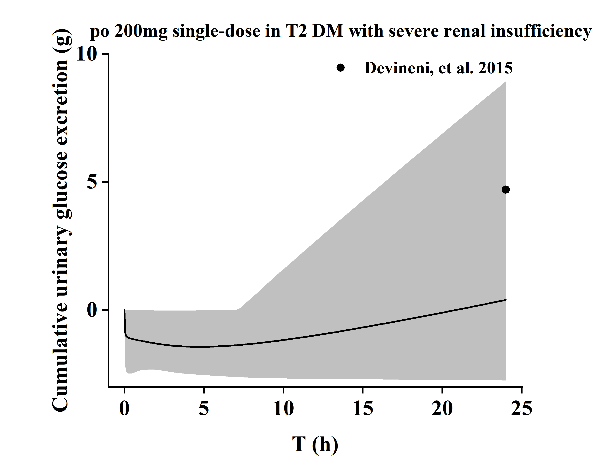


J

I


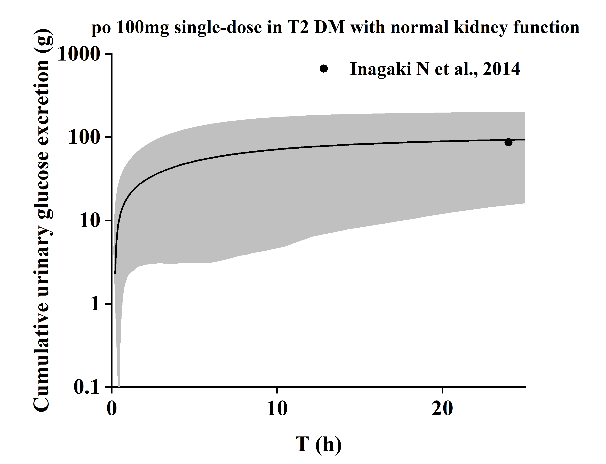

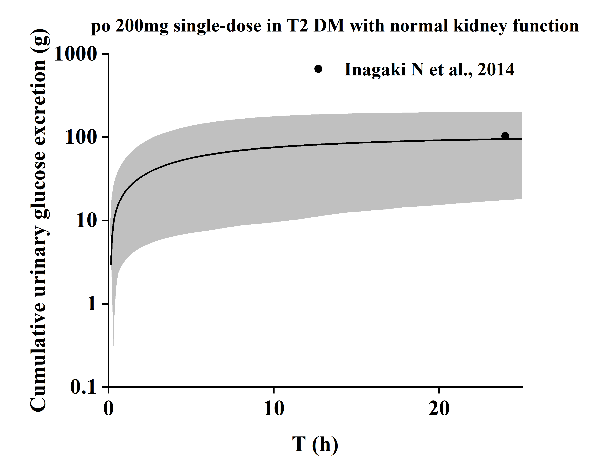


L

K


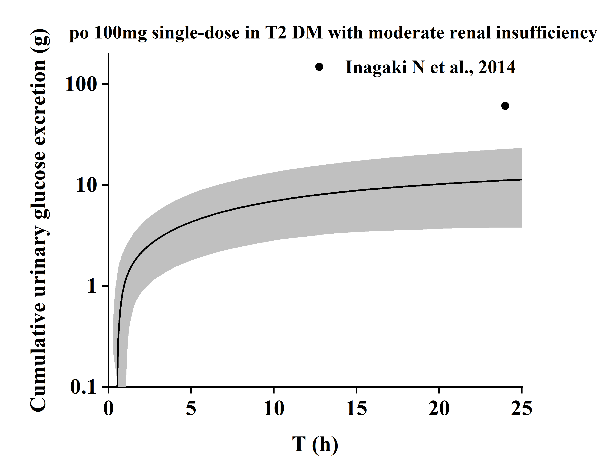

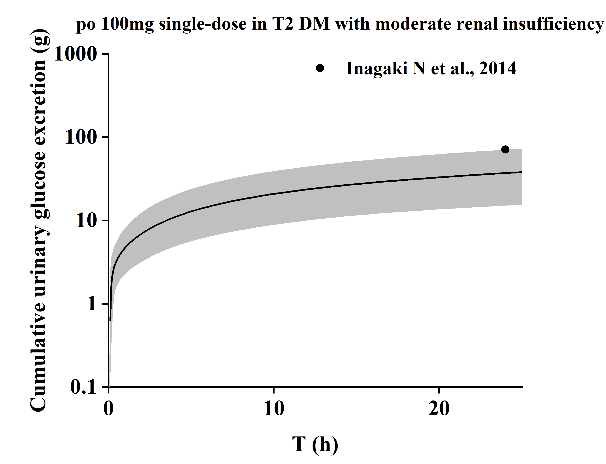


**Figure S5** Cumulative urinary glucose excretion after single and multiple oral doses of Canagliflozin in patients with normal renal function, renal insufficiency, and T2DM with renal insufficiency. Po: oral administration; qd.: once daily; T2DM patients with type 2 diabetes; grey shading was 90% confidence interval; black dots were measured values; (A-D) Clinical data for patients with normal renal function were obtained from Iijima et al., 2015 (Iijima et al., 2015); (E-H) Clinical data for patients with renal insufficiency were obtained from Devineni et al., 2015a (Devineni, Curtin, et al., 2015). (I-L) Clinical data of patients with renal insufficiency were obtained from Inagaki N et al., 2014 (Inagaki et al., 2014).

A

B


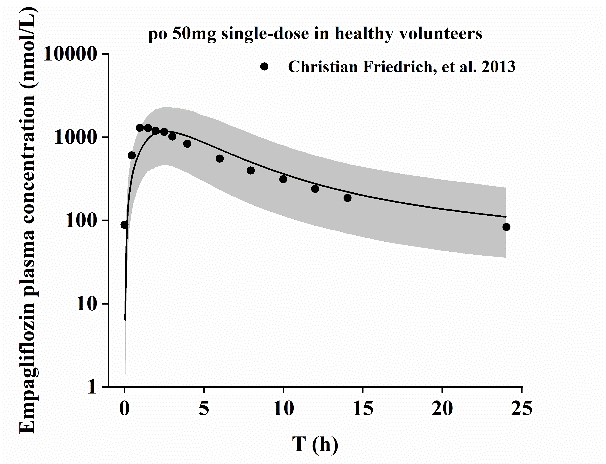

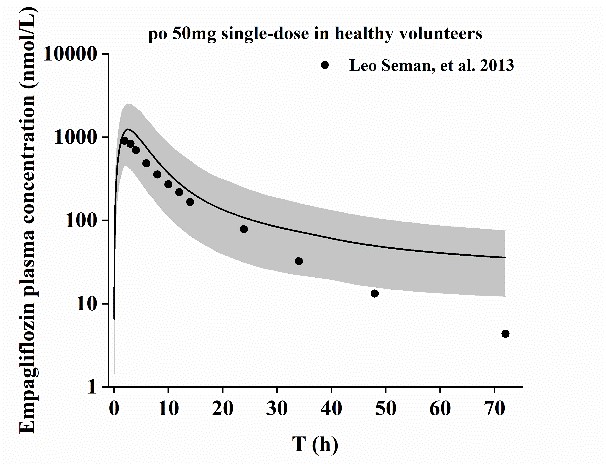


D

C


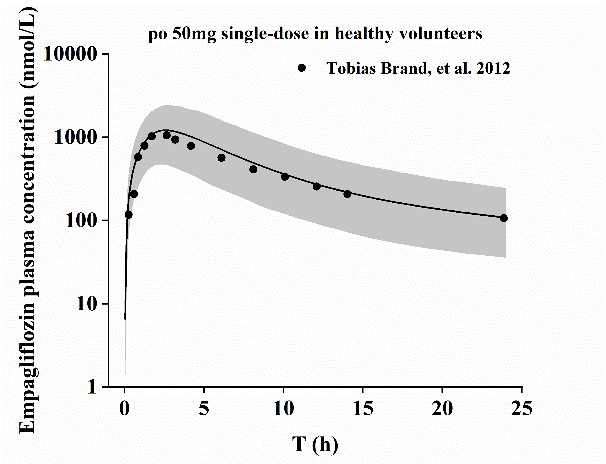

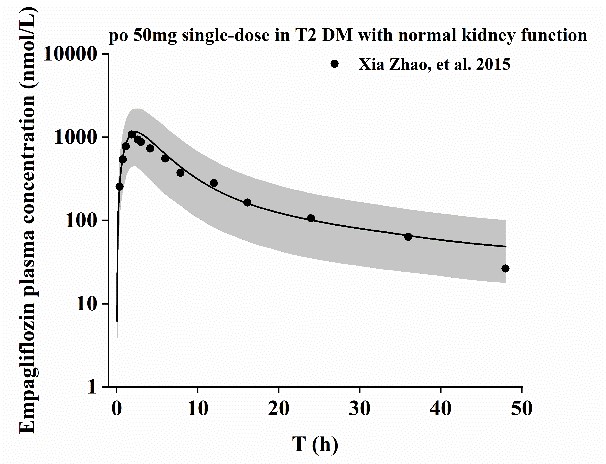


**Figure S6** Plasma concentration-time curve of single oral dose of Empagliflozin in healthy subjects and T2DM patients with normal renal function. Po: oral administration; T2DM patients with type 2 diabetes mellitus; grey shading was 90% confidence interval; black dots were measured values. (A) Healthy subject clinical data from Friedrich et al., 2013 (Friedrich et al., 2013); (B) Healthy subject clinical data from Seman et al., 2013 (Seman et al., 2013); (C) Healthy subject clinical data from Brand et al., 2012 (Brand et al., 2012); (D) Clinical data of patients with normal renal function were obtained from Zhao et al., 2015 (Zhao et al., 2015).

A

B


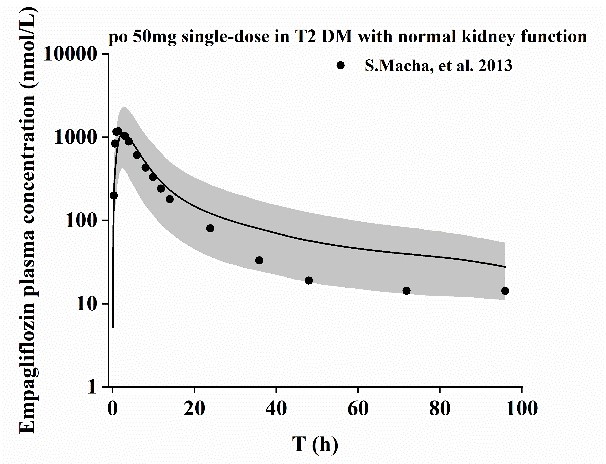

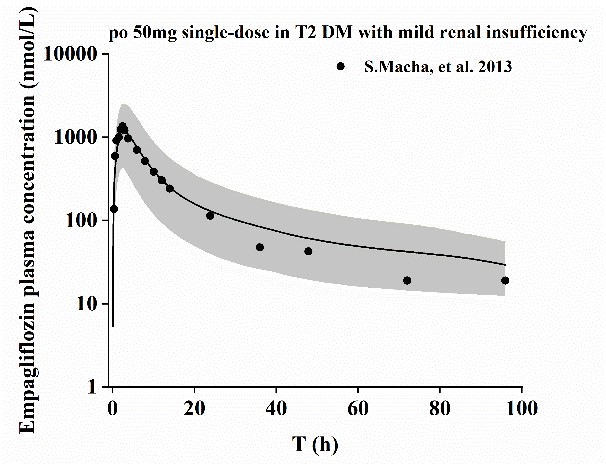


D

C


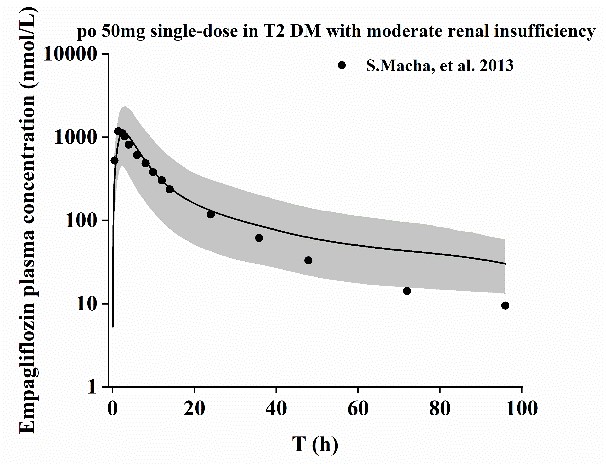

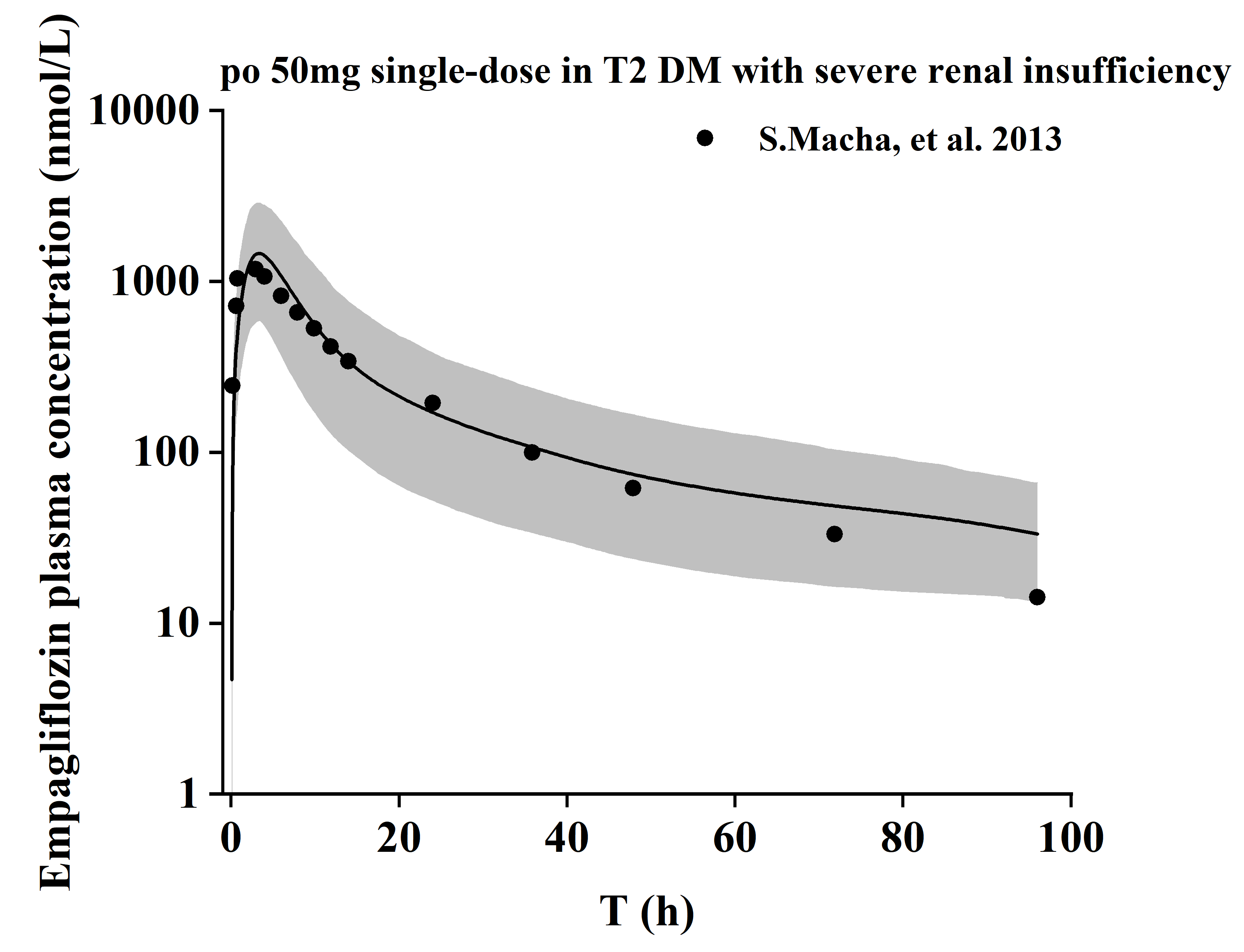


F

E


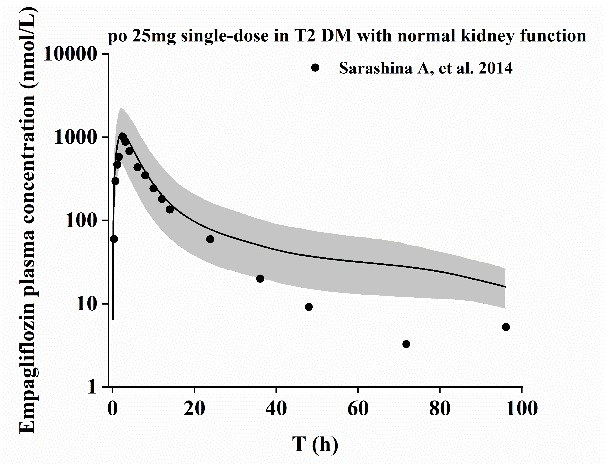

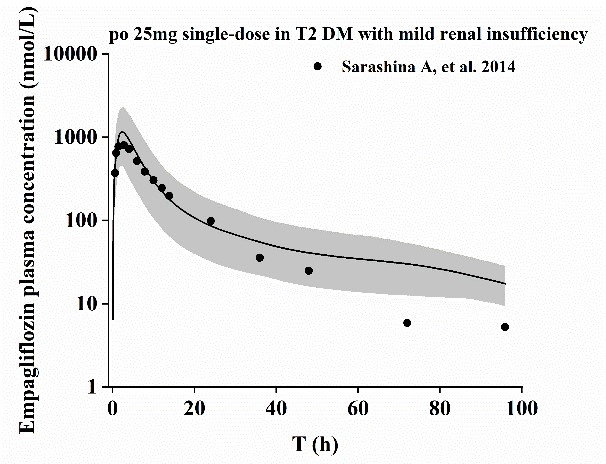


H

G


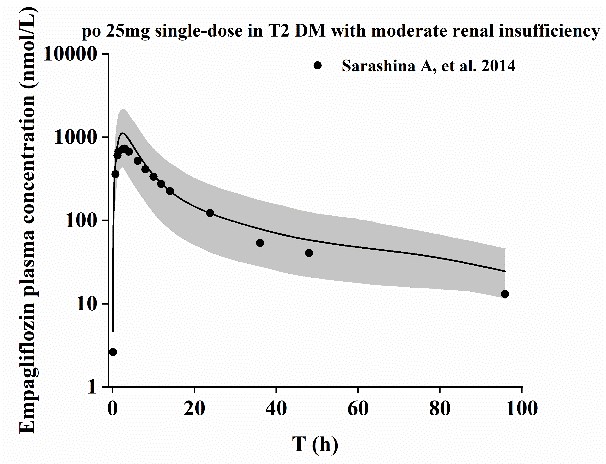

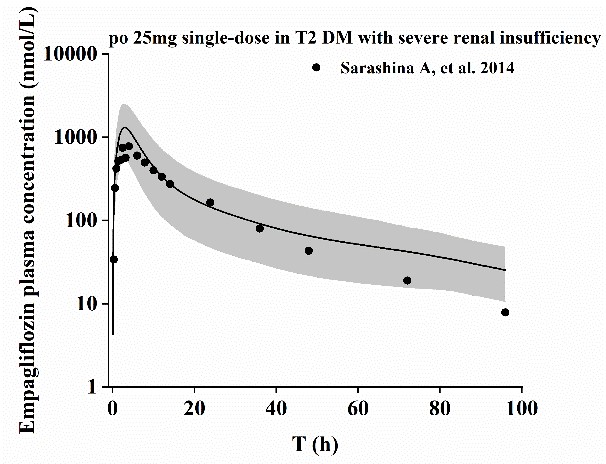


**Figure S7** Plasma concentration-time curve of single oral dose of Empagliflozin in T2DM patients with renal insufficiency. Po: oral administration; T2DM: type 2 diabetic patients; grey shading was 90% confidence interval; black dots were measured values. (A-D) Clinical data were obtained from Macha et al., 2014 (Macha et al., 2014); (E-H) Clinical data were obtained from Sarashina et al., 2014 (Sarashina et al., 2014).

A

B


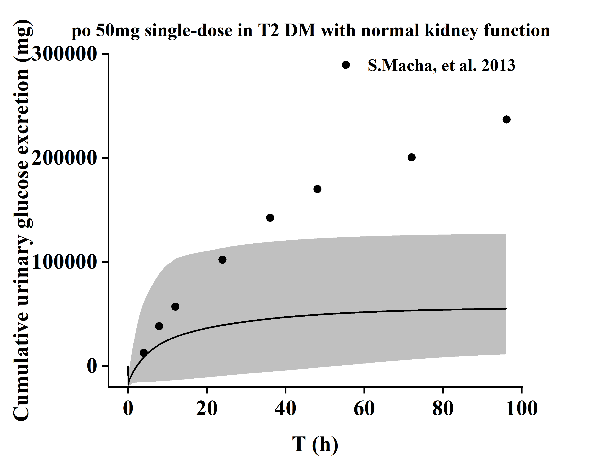

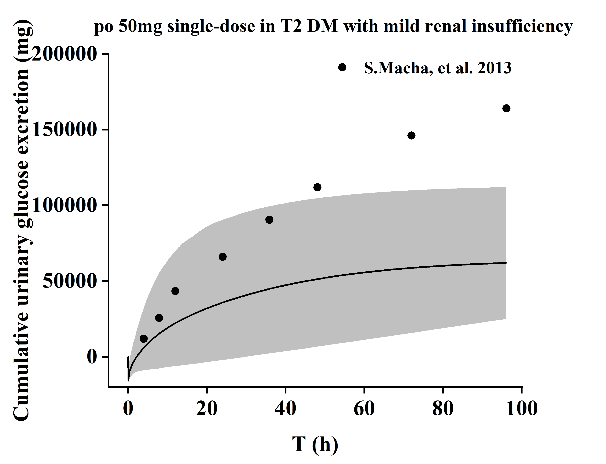


D

C


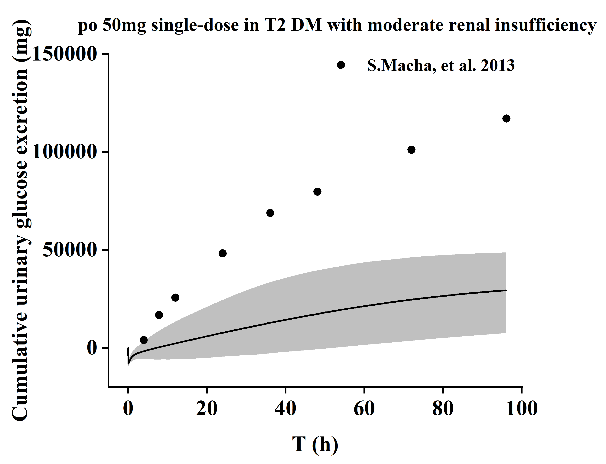

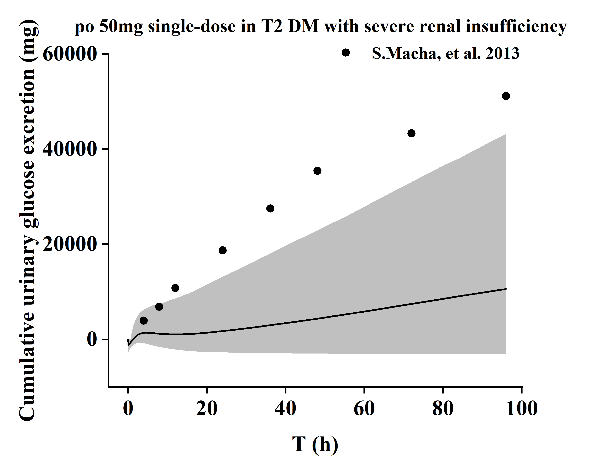


F

E


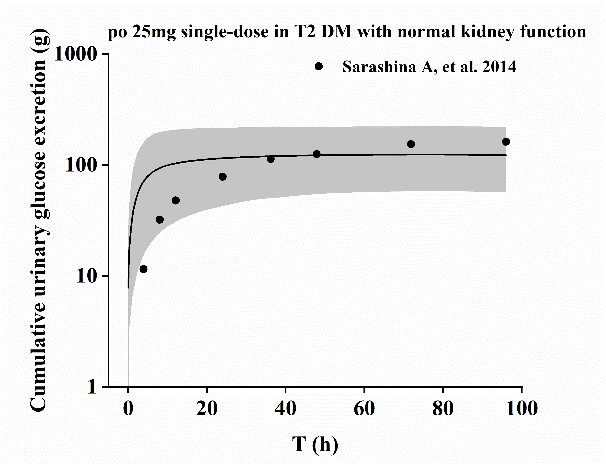

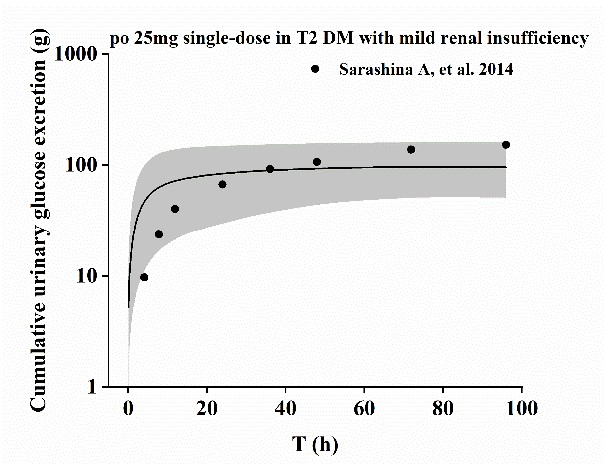


H

G


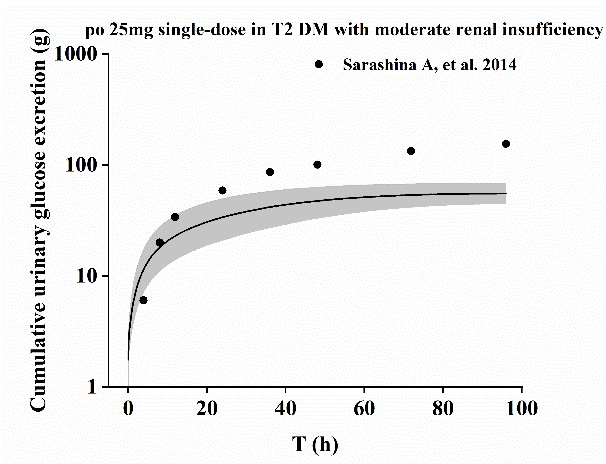

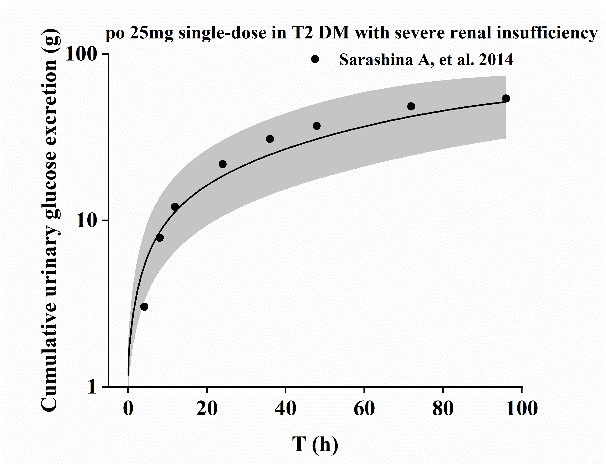


**Figure S8** Cumulative urinary glucose excretion after single oral dose of Empagliflozin in patients with renal insufficiency T2DM. Po: oral administration; T2DM: patients with type 2 diabetes mellitus; grey shading was 90% confidence interval; black dots were measured values. (A-D) Clinical data were obtained from Macha et al., 2014 (Macha et al., 2014); (E-H) Clinical data were obtained from Sarashina et al., 2014 (Sarashina et al., 2014).

A

B


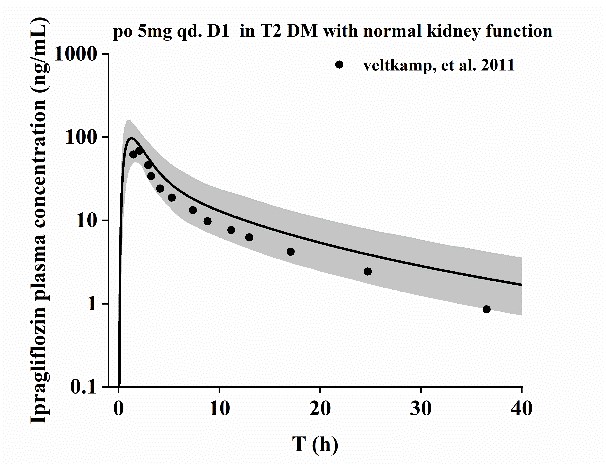

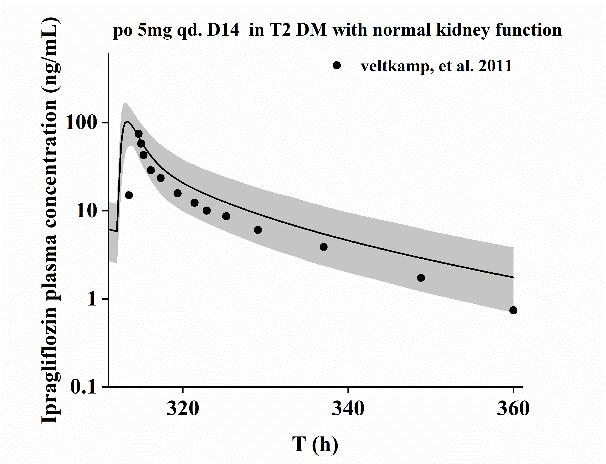


D

C


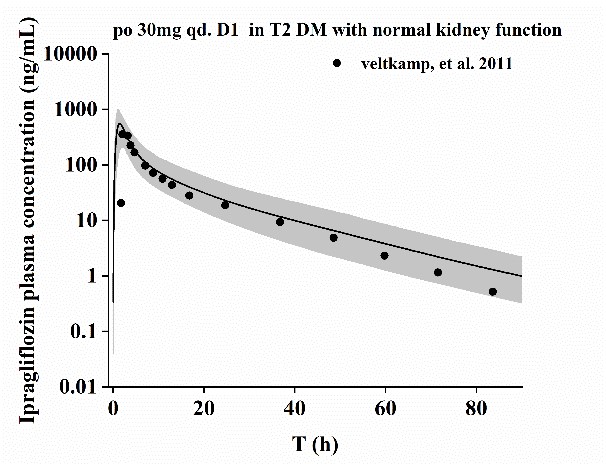

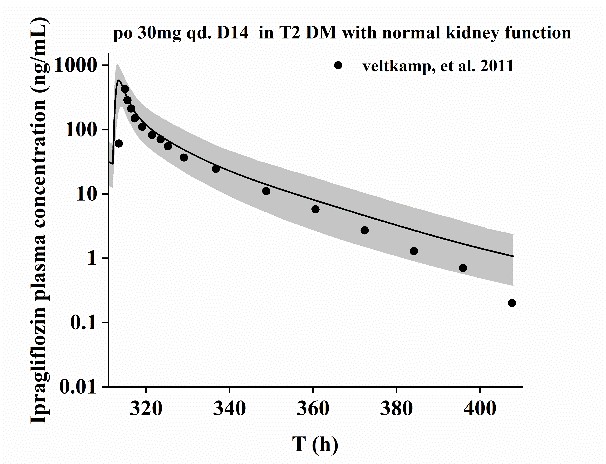


F

E


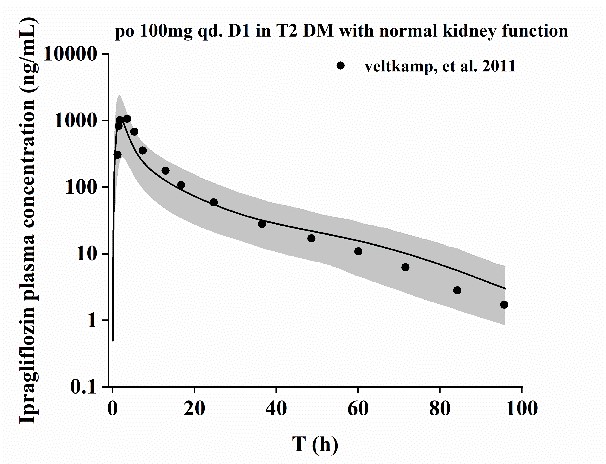

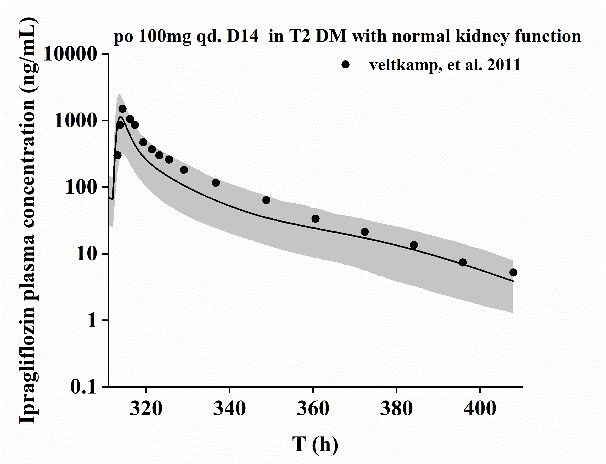


G


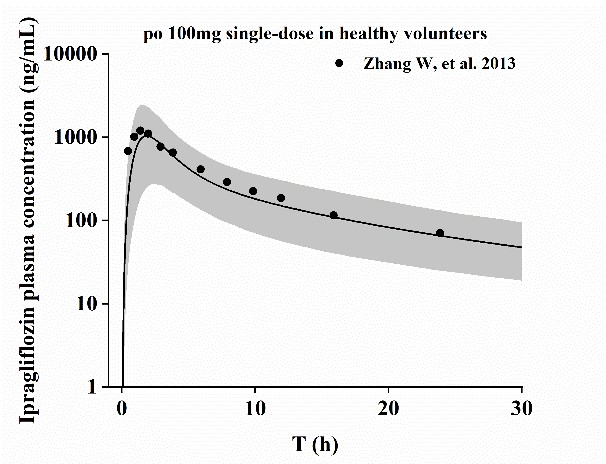


**Figure S9** Plasma concentration-time curves of single and multiple oral doses of Ipragliflozin in healthy subjects and T2DM patients with normal renal function. Po: oral administration; D1: day 1; D14: day 14; T2DM patients with type 2 diabetes mellitus; grey shading was 90% confidence interval; black dots were measured values. (A-F) Clinical data for patients with normal renal function from Veltkamp et al., 2011 (Veltkamp et al., 2011); (G) Clinical data for healthy subjects from Zhang et al., 2013 (Zhang et al., 2013).

A

B


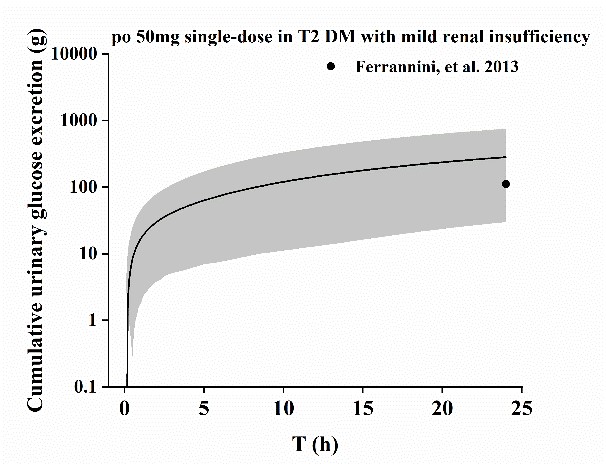

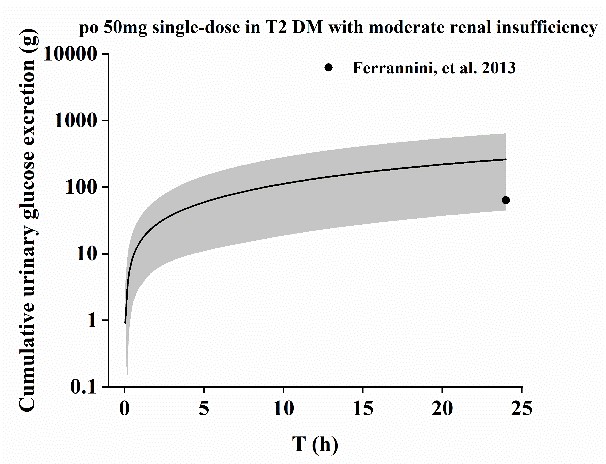


C


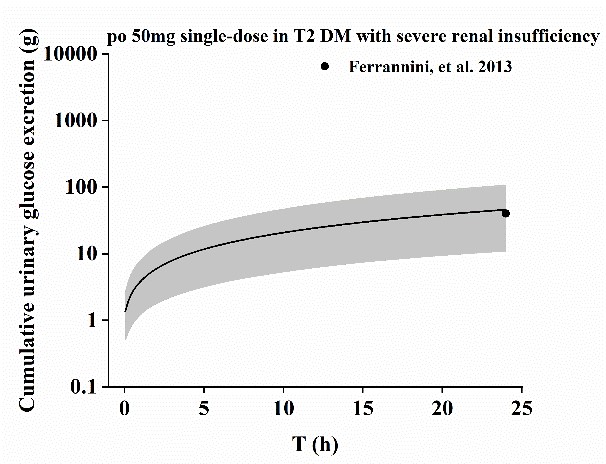


**Figure S10** Cumulative urinary glucose excretion after single oral dose of Ipragliflozin in patients with renal insufficiency T2DM. Po: oral administration; T2DM: patients with type 2 diabetes; grey shading was 90% confidence interval; black dots were measured values. (A-C) Clinical data were obtained from Ferrannini et al., 2013 (Ferrannini et al., 2013).





## Figure S11 Sensitivity analysis results of SGLT2 inhibitor PD model to three parameters of V^SGLT2^_max_, V^SGLT1^_max_ and Q_bladder_. （A） normal renal function T2DM patients; （B） mild renal insufficiency T2DM patients; （C） moderate renal insufficiency T2DM patients; （D） severe renal insufficiency T2DM patients.


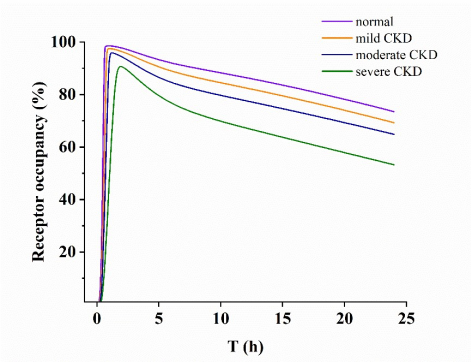


**Figure S12** The SGLT2 inhibitors (e.g., dapagliflozin) occupied SGLT2 transporter in T2DM patients with renal insufficiency.

A

B


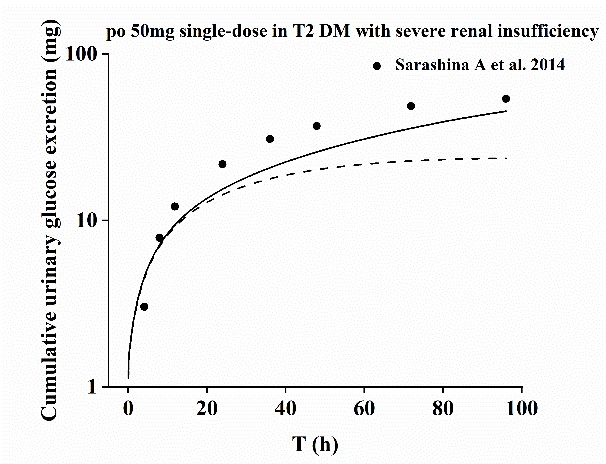

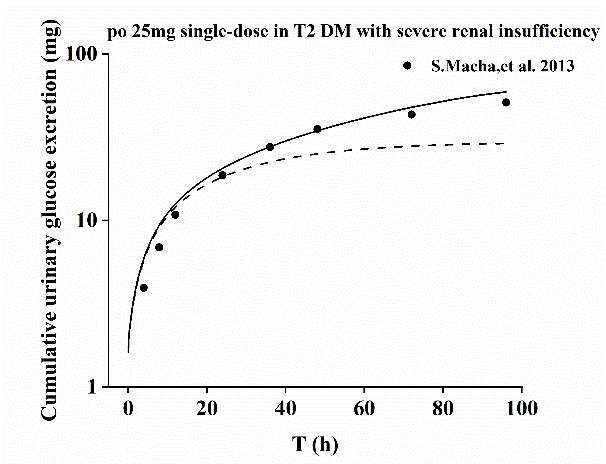


**Figure S13** Cumulative urinary glucose excretion in T2DM patients with severe renal insufficiency before and after optimization of Empagliflozin PD model. Po: oral administration; T2DM: patients with type 2 diabetes mellitus; gray shading was 90% confidence interval; black dots were measured values; black solid line represented the V^SGLT1^_max_ and V^SGLT2^_max_ values decreased by 60% and 90%, respectively; The black dashed line represented both V^SGLT1^_max_ and V^SGLT2^_max_ values decreased by 90%.

**References**

Brand, T., Macha, S., Mattheus, M., Pinnetti, S., & Woerle, H. J. (2012). Pharmacokinetics of empagliflozin, a sodium glucose cotransporter-2 (SGLT-2) inhibitor, coadministered with sitagliptin in healthy volunteers. *Adv Ther, 29*(10), 889-899. doi:10.1007/s12325-012-0055-3

Chen, L. H., & Leung, P. S. (2013). Inhibition of the sodium glucose co-transporter-2: its beneficial action and potential combination therapy for type 2 diabetes mellitus. *Diabetes Obes Metab, 15*(5), 392-402. doi:10.1111/dom.12064

Chen, X., Hu, P., Vaccaro, N., Polidori, D., Curtin, C. R., Stieltjes, H., et al. (2015). Pharmacokinetics, Pharmacodynamics, and Safety of Single-Dose Canagliflozin in Healthy Chinese Subjects. *Clin Ther, 37*(7), 1483-1492 e1481. doi:10.1016/j.clinthera.2015.04.015

Devineni, D., Curtin, C. R., Marbury, T. C., Smith, W., Vaccaro, N., Wexler, D., et al. (2015). Effect of hepatic or renal impairment on the pharmacokinetics of canagliflozin, a sodium glucose co-transporter 2 inhibitor. *Clin Ther, 37*(3), 610-628 e614. doi:10.1016/j.clinthera.2014.12.013

Devineni, D., Murphy, J., Wang, S. S., Stieltjes, H., Rothenberg, P., Scheers, E., & Mamidi, R. N. (2015). Absolute oral bioavailability and pharmacokinetics of canagliflozin: A microdose study in healthy participants. *Clin Pharmacol Drug Dev, 4*(4), 295-304. doi:10.1002/cpdd.162

Ferrannini, E., Veltkamp, S. A., Smulders, R. A., & Kadokura, T. (2013). Renal glucose handling: impact of chronic kidney disease and sodium-glucose cotransporter 2 inhibition in patients with type 2 diabetes. *Diabetes Care, 36*(5), 1260-1265. doi:10.2337/dc12-1503

Friedrich, C., Metzmann, K., Rose, P., Mattheus, M., Pinnetti, S., & Woerle, H. J. (2013). A randomized, open-label, crossover study to evaluate the pharmacokinetics of empagliflozin and linagliptin after coadministration in healthy male volunteers. *Clin Ther, 35*(1), A33-42. doi:10.1016/j.clinthera.2012.12.002

Iijima, H., Kifuji, T., Maruyama, N., & Inagaki, N. (2015). Pharmacokinetics, Pharmacodynamics, and Safety of Canagliflozin in Japanese Patients with Type 2 Diabetes Mellitus. *Adv Ther, 32*(8), 768-782. doi:10.1007/s12325-015-0234-0

Inagaki, N., Kondo, K., Yoshinari, T., Ishii, M., Sakai, M., Kuki, H., & Furihata, K. (2014). Pharmacokinetic and pharmacodynamic profiles of canagliflozin in Japanese patients with type 2 diabetes mellitus and moderate renal impairment. *Clin Drug Investig, 34*(10), 731-742. doi:10.1007/s40261-014-0226-x

Kasichayanula, S., Chang, M., Hasegawa, M., Liu, X., Yamahira, N., LaCreta, F. P., et al. (2011). Pharmacokinetics and pharmacodynamics of dapagliflozin, a novel selective inhibitor of sodium-glucose co-transporter type 2, in Japanese subjects without and with type 2 diabetes mellitus. *Diabetes Obes Metab, 13*(4), 357-365. doi:10.1111/j.1463-1326.2011.01359.x

Kasichayanula, S., Liu, X., Lacreta, F., Griffen, S. C., & Boulton, D. W. (2014). Clinical pharmacokinetics and pharmacodynamics of dapagliflozin, a selective inhibitor of sodium-glucose co-transporter type 2. *Clin Pharmacokinet, 53*(1), 17-27. doi:10.1007/s40262-013-0104-3

Kim, S., Heo, N. J., Jung, J. Y., Son, M. J., Jang, H. R., Lee, J. W., et al. (2010). Changes in the sodium and potassium transporters in the course of chronic renal failure. *Nephron Physiol, 115*(4), p31-41. doi:10.1159/000314542

Lim, Y. J., Sidor, N. A., Tonial, N. C., Che, A., & Urquhart, B. L. (2021). Uremic Toxins in the Progression of Chronic Kidney Disease and Cardiovascular Disease: Mechanisms and Therapeutic Targets. *Toxins (Basel), 13*(2). doi:10.3390/toxins13020142

Macha, S., Mattheus, M., Halabi, A., Pinnetti, S., Woerle, H. J., & Broedl, U. C. (2014). Pharmacokinetics, pharmacodynamics and safety of empagliflozin, a sodium glucose cotransporter 2 (SGLT2) inhibitor, in subjects with renal impairment. *Diabetes Obes Metab, 16*(3), 215-222. doi:10.1111/dom.12182

Mori, K., Saito, R., Nakamaru, Y., Shimizu, M., & Yamazaki, H. (2016). Physiologically based pharmacokinetic-pharmacodynamic modeling to predict concentrations and actions of sodium-dependent glucose transporter 2 inhibitor canagliflozin in human intestines and renal tubules. *Biopharm Drug Dispos, 37*(8), 491-506. doi:10.1002/bdd.2040

Nakamura, N., Masuda, S., Takahashi, K., Saito, H., Okuda, M., & Inui, K. (2004). Decreased expression of glucose and peptide transporters in rat remnant kidney. *Drug Metab Pharmacokinet, 19*(1), 41-47. doi:10.2133/dmpk.19.41

Sarashina, A., Ueki, K., Sasaki, T., Tanaka, Y., Koiwai, K., Sakamoto, W., et al. (2014). Effect of renal impairment on the pharmacokinetics, pharmacodynamics, and safety of empagliflozin, a sodium glucose cotransporter 2 inhibitor, in Japanese patients with type 2 diabetes mellitus. *Clin Ther, 36*(11), 1606-1615. doi:10.1016/j.clinthera.2014.08.001

Seman, L., Macha, S., Nehmiz, G., Simons, G., Ren, B., Pinnetti, S., et al. (2013). Empagliflozin (BI 10773), a Potent and Selective SGLT2 Inhibitor, Induces Dose-Dependent Glucosuria in Healthy Subjects. *Clin Pharmacol Drug Dev, 2*(2), 152-161. doi:10.1002/cpdd.16

Vallon, V., & Thomson, S. C. (2020). The tubular hypothesis of nephron filtration and diabetic kidney disease. *Nat Rev Nephrol, 16*(6), 317-336. doi:10.1038/s41581-020-0256-y

Veltkamp, S. A., Kadokura, T., Krauwinkel, W. J., & Smulders, R. A. (2011). Effect of Ipragliflozin (ASP1941), a novel selective sodium-dependent glucose co-transporter 2 inhibitor, on urinary glucose excretion in healthy subjects. *Clin Drug Investig, 31*(12), 839-851. doi:10.1007/BF03256922

Vrhovac, I., Balen Eror, D., Klessen, D., Burger, C., Breljak, D., Kraus, O., et al. (2015). Localizations of Na(+)-D-glucose cotransporters SGLT1 and SGLT2 in human kidney and of SGLT1 in human small intestine, liver, lung, and heart. *Pflugers Arch, 467*(9), 1881-1898. doi:10.1007/s00424-014-1619-7

Wang, Z., Wang, G., & Ren, J. (2022). Using a Mathematical Modeling To Simulate Pharmacokinetics and Urinary Glucose Excretion of Luseogliflozin and Explore the Role of SGLT1/2 in Renal Glucose Reabsorption. *ACS Omega, 7*(51), 48427-48437. doi:10.1021/acsomega.2c06483

Yakovleva, T., Sokolov, V., Chu, L., Tang, W., Greasley, P. J., Peilot Sjogren, H., et al. (2019). Comparison of the urinary glucose excretion contributions of SGLT2 and SGLT1: A quantitative systems pharmacology analysis in healthy individuals and patients with type 2 diabetes treated with SGLT2 inhibitors. *Diabetes Obes Metab, 21*(12), 2684-2693. doi:10.1111/dom.13858

Yang, Y., & Xu, G. (2022). Update on Pathogenesis of Glomerular Hyperfiltration in Early Diabetic Kidney Disease. *Front Endocrinol (Lausanne), 13*, 872918. doi:10.3389/fendo.2022.872918

Zhang, W., Krauwinkel, W. J., Keirns, J., Townsend, R. W., Lasseter, K. C., Plumb, L., et al. (2013). The effect of moderate hepatic impairment on the pharmacokinetics of ipragliflozin, a novel sodium glucose co-transporter 2 (SGLT2) inhibitor. *Clin Drug Investig, 33*(7), 489-496. doi:10.1007/s40261-013-0089-6

Zhao, X., Cui, Y., Zhao, S., Lang, B., Broedl, U. C., Salsali, A., et al. (2015). Pharmacokinetic and Pharmacodynamic Properties and Tolerability of Single- and multiple-dose Once-daily Empagliflozin, a Sodium Glucose Cotransporter 2 Inhibitor, in Chinese Patients With Type 2 Diabetes Mellitus. *Clin Ther, 37*(7), 1493-1502. doi:10.1016/j.clinthera.2015.05.001
